# Rare *CASP6*N73T variant associated with hippocampal volume exhibits decreased proteolytic activity, synaptic transmission defect, and neurodegeneration

**DOI:** 10.1038/s41598-021-91367-0

**Published:** 2021-06-16

**Authors:** Libin Zhou, Kwangsik Nho, Maria G. Haddad, Nicole Cherepacha, Agne Tubeleviciute-Aydin, Andy P. Tsai, Andrew J. Saykin, P. Jesper Sjöström, Andrea C. LeBlanc

**Affiliations:** 1 Lady Davis Institute for Medical Research at Jewish General Hospital, Montréal, QC Canada; 2 Department of Anatomy and Cell Biology, McGill University, Montréal, QC Canada; 3 Department of Radiology and Imaging Sciences and Indiana Alzheimer’s Disease Research Center, Indiana University School of Medicine, Indianapolis, IN USA; 4 Centre for Research in Neuroscience, the BRaIN Program, Department of Medicine, and Department of Neurology and Neurosurgery, The Research Institute of the McGill University Health Centre, Montreal General Hospital, McGill University, 1650 Cedar Avenue Montreal, Montréal, QC H3G 1A4 Canada; 5 Department of Neurology and Neurosurgery, McGill University, Montréal, QC Canada; 6 Stark Neurosciences Research Institute, Indiana University School of Medicine, Indianapolis, IN USA

**Keywords:** Biochemistry, Genetics, Neuroscience

## Abstract

Caspase-6 (Casp6) is implicated in Alzheimer disease (AD) cognitive impairment and pathology. Hippocampal atrophy is associated with cognitive impairment in AD. Here, a rare functional exonic missense *CASP6* single nucleotide polymorphism (SNP), causing the substitution of asparagine with threonine at amino acid 73 in Casp6 (Casp6N73T), was associated with hippocampal subfield CA1 volume preservation. Compared to wild type Casp6 (Casp6WT), recombinant Casp6N73T altered Casp6 proteolysis of natural substrates Lamin A/C and α-Tubulin, but did not alter cleavage of the Ac-VEID-AFC Casp6 peptide substrate. Casp6N73T-transfected HEK293T cells showed elevated Casp6 mRNA levels similar to Casp6WT-transfected cells, but, in contrast to Casp6WT, did not accumulate active Casp6 subunits nor show increased Casp6 enzymatic activity. Electrophysiological and morphological assessments showed that Casp6N73T recombinant protein caused less neurofunctional damage and neurodegeneration in hippocampal CA1 pyramidal neurons than Casp6WT. Lastly, *CASP6* mRNA levels were increased in several AD brain regions confirming the implication of Casp6 in AD. These studies suggest that the rare Casp6N73T variant may protect against hippocampal atrophy due to its altered catalysis of natural protein substrates and intracellular instability thus leading to less Casp6-mediated damage to neuronal structure and function.

## Introduction

Caspase-6 (Casp6), a cysteine protease that cleaves its protein substrates after an aspartic acid residue, is associated with Alzheimer disease (AD) pathogenesis. Active Casp6, Casp6-cleaved Tau (Tau∆Casp6), Casp6-cleaved α-Tubulin (Tub∆Casp6), and Casp6-cleaved valosin containing protein p97 (p97∆Casp6) are detected in neurofibrillary tangles (NFT), neuritic plaques and neuropil threads of sporadic and familial AD and mild cognitively impaired (MCI) brains, but absent in young control brains^1–4^. Active Casp6 and Tau∆Casp6 are observed in pre- and mature NFT, but only Tau∆Casp6 is still present in ghost tangles^5,6^. Surprisingly, in aged non-cognitively impaired (NCI) brains, Casp6 activity is detected uniquely in the inter-neuronally connected entorhinal cortex (ERC) and hippocampal Cornu Ammonis 1 (CA1)^1,5,7^, the first areas of the brain presenting with NFT according to Braak staging^8,9^. Furthermore, active Casp6 is detected in AD pathologies of the anterior olfactory bulb, whose neurons project their axons into the ERC^10^. Therefore, Casp6 activity is an early spatial–temporal event in AD pathogenesis.

Casp6 activity is associated with age-dependent cognitive impairment. In aged NCI individuals, high levels of Casp6 correlate with global cognitive scores and predict lower episodic and semantic memory performance, the first types of memories impaired in AD^1,7,11^. Casp6 activity in the anterior olfactory bulb is associated with lower global cognitive scores, mini mental state exam (MMSE) scores, and episodic and semantic memories^10^. Cerebrospinal fluid Tau∆Casp6 levels reflect human brain Tau∆Casp6 levels, and correlate with AD severity, and global cognitive, MMSE, episodic, semantic and working memory scores^12^. Furthermore, expression of self-activated human Casp6 in mouse CA1 neurons is sufficient to induce age-dependent episodic and spatial memory impairment^11^.

Active Casp6 is associated with selective neuronal degeneration, but not necessarily cell death. Casp6 activation in HEK293T cells does not induce cell death^13,14^. Microinjection of recombinant active Casp6 in human neurons in primary cultures induces a dose-dependent and protracted type of cell death^15^. Similarly, overexpression of wild type or mutant forms of amyloid precursor protein (APP) associated with familial AD induces Casp6-dependent, but amyloid beta peptide (Aβ) independent, neurodegeneration in human neurons^16^. This eventually leads to neuronal cell death that is Aβ dependent. Nerve growth factor (NGF)-deprived mouse primary dorsal root ganglia and sympathetic neuron cultures leads to compartmentalized Casp6-dependent axonal degeneration and Caspase-3 (Casp3)-dependent cell death^17–19^. In AD brains, active Casp6-immunopositive neurons do not show apoptotic features and active Casp3 immunoreactivity is sparse^6,20,21^. Furthermore, transgenic expression of a self-activated human Casp6 in mouse brains causes hippocampal CA1 neurodegeneration, defined here as the loss of neuronal structure or function, in the absence of significant neuronal loss^11,22^. Consistently, microinjected active Casp6 causes neuronal degeneration and impairs synaptic transmission in CA1 pyramidal neurons, but not in medium spiny neurons^22^.

Casp6 cleaves many protein substrates such as α-Tubulin, Tau, α-Actinin-4, Drebrin, Glial fibrillary acidic protein, Spinophilin, and Vimentin which are critical for neuronal structure and function and synaptic plasticity^2,5,23,24^. Casp6-cleaved valosin containing protein p97 impairs ubiquitin proteasome system-mediated protein degradation^3^. Ubiquitin ligases of mouse double minute 2 homolog, ubiquitin fusion degradation 2, and developmentally down-regulated protein 4, proteins involved in cellular protein turnover are also Casp6 substrates^25,26^. Furthermore, Casp6 cleaves APP, Presenilin 1 and 2, and Huntingtin, proteins involved in neurodegenerative diseases^27–29^. These neuronal substrates reflect Casp6 potential function in neurodegeneration.

Casp6 is activated by overexpression and proteolytic processing. Human Casp6 is expressed as an inactive zymogen from the *CASP6* gene^30^ and the level of Casp6 protein is relatively low in normal human brain^31^. Prokaryotic and eukaryotic overexpression of Casp6 is sufficient for self-proteolytic activation^14,32,33^. *CASP6* transcriptional regulation has not been extensively investigated, but p53 is a CASP6 transcription regulator in neurons and astrocytes^34–36^. p53 levels are enhanced in familial AD neurons^37^, indicating a mechanism for increased *CASP6* gene expression. The Casp6 active site consists of catalytic cysteine 163 (C163) surrounded by four binding pockets for substrate selectivity^38^. Casp6 maturation requires three cleavage events at D23, D179, and D193 to remove the pro-domain and the inter-subunit linker between a large subunit (LS) and a small subunit (SS), so a small β-sheet structure 190TEVD193 in the inter-subunit linker occupying the active site is removed and catalytic C163 is exposed^33^. Proteolytic processing allows the correct conformation of exosites and allosteric sites important for the regulation of catalytic specificity and efficiency^39,40^. Casp6 can be processed and activated by itself at high concentration^14,32^, by Casp3 in apoptotic cells^41^, or by Caspase-1 in stressed primary human neurons^42,43^. Interestingly, p53 also transcriptionally regulates *CASP1* gene expression^44^, which can also self-activate, indicating possible co-regulated expression of *CASP1* and *CASP6* genes.

Many genetic risk factors have been identified for AD, but no genetic association between *CASP6* and AD has been reported. Here, we used SKAT-O analysis^45^ to identify whether rare functional exonic *CASP6* variants associate with human brain volumes, determined by MRI scans in aged individuals. A rare variant, Casp6N73T, associated with hippocampal subfield CA1 volumes in ADNI. Recombinant Casp6N73T exhibited altered proteolysis of natural protein substrates compared to wild type Casp6 (Casp6WT), limited enzymatic activity and stability in cell culture, and eliminated Casp6-mediated dysfunction and degeneration in hippocampal CA1 pyramidal neurons. These results suggest that Casp6N73T may provide a protective effect against hippocampal atrophy compared to Casp6WT.

## Results

### Genetic association between the Caspase-6 gene (*CASP6*) and hippocampal CA1 volume in AD

From the Alzheimer’s Disease Neuroimaging Initiative (ADNI) whole genome sequencing (WGS) data (n = 757), only one functional exonic missense SNP (rs141550898) with a minor allele frequency < 0.05 was identified within CASP6. We performed SKAT-O of rs141550898 with CA1 volumes with age, sex, year of education, MRI field strength, and intracranial volume as covariates. One individual (male, early MCI, APOE ε3/ε3) was heterozygous for this variant. The optimized sequence kernel association test (SKAT-O) yielded a significant association of rs141550898 in *CASP6* with hippocampal CA1 subfield volume (p = 0.0146). The rs141550898 SNP in which AAT is replaced by ACT causes a substitution of asparagine (N) for threonine (T) at amino acid 73 of Casp6, thus generating Casp6N73T. The participant carrying Casp6N73T has maintained its early MCI status for at least 5 year based on his last visit, raising the possibility that Casp6N73T may slow down hippocampal CA1 atrophy and AD progression.

### Gene expression of *CASP6* and *CASP1*

**Figure 1:**
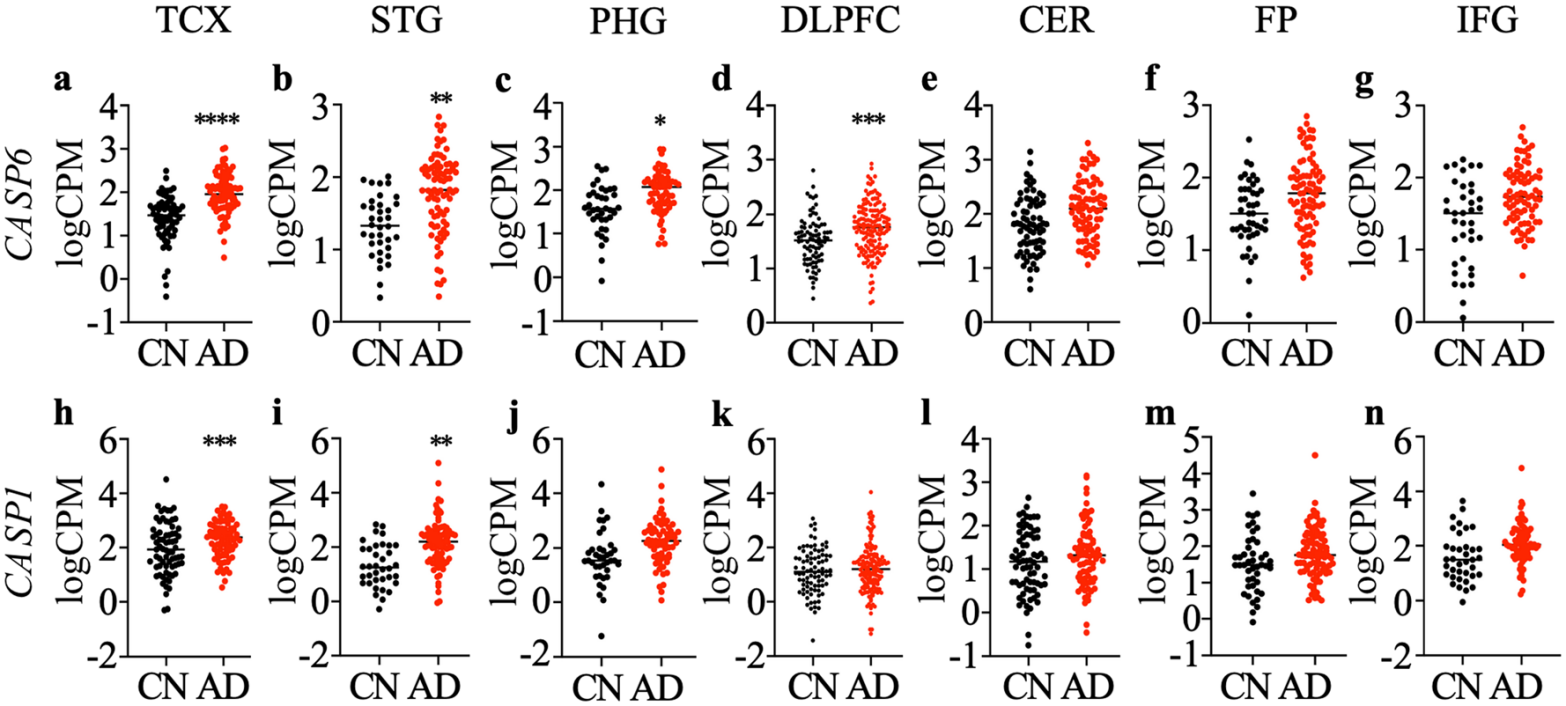
Differential expression of *CASP6* and *CASP1* in Alzheimer disease (AD) and cognitively normal (CN) brains. The logarithm of read counts per million total reads (logCPM) values for *CASP6* mRNA (**a**–**g**) and *CASP1* mRNA (**h**–**n**) generated from RNA-Seq data for AD and CN temporal cortex (TCX; **a**,**h**), superior temporal gyrus (STG; **b**,**i**), para-hippocampal gyrus (PHG; **c**,**j**), dorsolateral prefrontal cortex (DLPFC; **d**,**k**), cerebellum (CER; **e**,**l**), frontal pole (FP; **f**,**m**) and inferior frontal gyrus (IFG; **g**,**n**). Each dot represents data from one individual and the horizontal bar denotes the mean. Statistical evaluations were done with the R package limma between AD and CN. *P < 0.05, **p < 0.01, ***P < 0.001, ****p < 0.0001.

To confirm the implication of *CASP1* and *CASP6* in the brains of individuals assessed in the ADNI cohort, *CASP1* and *CASP6* mRNA levels were evaluated. The number, age, sex, mean age at death, and apolipoprotein *APO* E genotype of the participants used for differential gene expression analysis in each brain region are summarized in Table 1. The RNA integrity number (RIN) where 10 represents intact RNA and 1 completely degraded RNA ranged from 6.3 to 8.7. Gene expression association analysis using RNA-Seq data generated from temporal cortex (TCX), para-hippocampal gyrus (PHG), inferior frontal gyrus (IFG), superior temporal gyrus (STG), frontal pole (FP), dorsolateral prefrontal cortex (DLPFC), and cerebellum (CER) identified that *CASP6* mRNA was up-regulated in the TCX (Fig. 1a), STG (Fig. 1b), PHG (Fig. 1c), and DLPFC (Fig. 1d) of AD compared to cognitively normal older adults. Levels in the CER (Fig. 1e), FP (Fig. 1f), and IFG (Fig. 1g) were not significantly different. Casp6 is activated by Caspase-1 (Casp1), therefore *CASP1* mRNA levels were also evaluated. *CASP1* mRNA levels were significantly up-regulated in AD TCX (Fig. 1h) and STG (Fig. 1i), but not in PHG (Fig. 1j), DLPFC (Fig. 1k), CER (Fig. 1l), FP (Fig. 1m), or IFG (Fig. 1n), compared to cognitively normal older adults.

**Table 1:** Demographic and clinical characteristics of cognitively normal (CN) and Alzheimer disease (AD) participants for different brain region analysed.

Brain region	TCX (Mayo)		PHG (MSBB)		IFG (MSBB)		STG (MSBB)		FP (MSBB)		DLPFC (ROSMAP)		CER (Mayo)	
Diagnosis	CN	AD	CN	AD	CN	AD	CN	AD	CN	AD	CN	AD	CN	AD
Number	71	80	16	78	18	102	21	98	22	111	86	155	72	79
Sex (F/M)	35/36	49/31	11/5	53/25	12/6	69/33	16/5	65/33	16/6	75/36	47/39	109/46	35/37	47/32
Age at death (SD), years	82.7 (8.5)	82.6 (7.7)	83.5 (8.9)	85.5 (6.1)	83.2 (8.6)	85.4 (6.0)	83.8 (8.1)	84.5 (6.7)	83.1 (7.7)	85.4 (5.9)	83.4 (5.9)	88.2 (3.1)	82.3 (8.3)	82.5 (7.7)
RIN (SD)	7.7 (1.0)	8.6 (0.6)	7.1 (0.9)	6.5 (0.9)	8.7 (1.4)	8.0 (1.8)	6.4 (1.0)	6.3 (0.9)	6.8 (0.9)	6.8 (0.9)	7.3 (1.0)	6.9 (0.9)	7.7 (1.0)	8.4 (0.7)
APOE genotype (ε4 + /ε4-)	62/9	38/42	14/2	57/21	16/2	72/30	17/4	65/33	19/3	70/41	77/9	91/64	62/10	38/41

*TCX* temporal cortex, *PHG* para-hippocampal gyrus, *IFG* inferior frontal gyrus, *STG* superior temporal gyrus, *FP* frontal pole, *DLPFC* dorsolateral prefrontal cortex, *CER* cerebellum, *RIN *RNA integrity number.

### Recombinant Caspase-6 N73T (Casp6N73T) zymogen showed similar self-processing as Caspase-6 wild type (Casp6WT) zymogen

**Figure 2:**
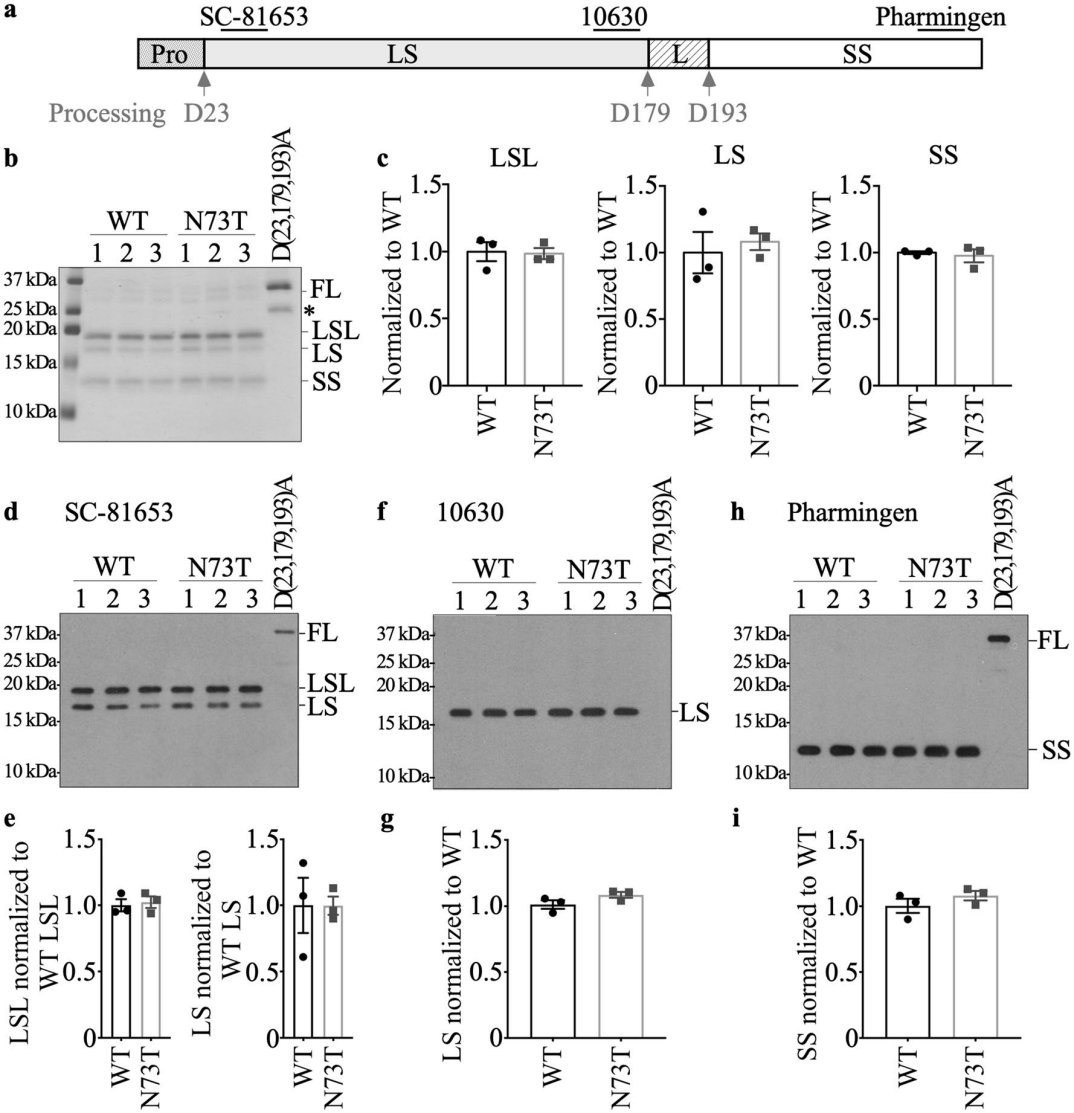
Self-processing of recombinant Casp6N73T zymogen. (**a**) Schematic diagram of recombinant human Casp6 zymogen showing the subunits and epitopes recognized by antibodies. The epitope for the SC-81635 antibody is unknown but located in the large subunit (LS), neoepitope for 10630 antisera detects the C-terminus of the LS of Casp6, and epitope for the Pharmingen antibody is G271-K285. *Pro* pro-domain, *LS* large subunit, *L* linker, *SS* small subunit. (**b**) Coomassie blue stain of 2 µg purified recombinant Casp6WT and Casp6N73T zymogens processed into LSL, LS and SS subunits. The unprocessed Casp6D(23,179,193)A was used as a control for the FL form. *LSL* large subunit with the linker, *FL* full length. * indicates unspecific band. (**c**) Quantification of the density of LSL, LS or SS normalized to density levels of Casp6WT from Coomassie blue stained gels. (**d,f,h**) Western blot of Casp6WT, Casp6N73T, and Casp6D(23,179,193)A with SC-81635 antibody anti-LS (**d**)**,** with neoepitope antisera 10630 anti cleaved LS (**f),** and with Pharmingen antibody anti-SS **(h)**. Quantification of (**e)** LSL and LS from (d), **(g)** LS from (f), and (**i)** SS from (h). Data represents mean and s.e.m of 3 independent experiments. No statistical differences were found by unpaired student’s test.

To observe the self-processing of Caspase-6 N73T (Casp6N73T) zymogen, recombinant protein from three independent clones expressing Casp6N73T zymogen were compared with Caspase-6 wild type (Casp6WT) zymogen and catalytically inactive triple mutant Casp6D(23,179,193)A. The Casp6WT zymogen undergoes self-processing at D23, D179, and D193 to remove the pro-domain and the inter-subunit linker, which allows the formation of active Casp6 consisting of two large subunits (LS) and two small subunits (SS) (Fig. 2a). Purified recombinant Casp6N73T and Casp6WT zymogens both generated the expected small subunit (SS) and large subunit with (LSL) or without (LS) inter-subunit linker. Casp6WT and Casp6N73T zymogens generated more LSL than LS (Fig. 2b) indicating preferred self-cleavage at D193, as previously observed with Casp6WT^14,33,46^. The catalytically inactive Casp6D(23,179,193)A was not processed, as expected, and migrated as full-length (FL) Casp6 at 34 kDa. The Casp6N73T zymogen generated LSL, LS, and SS at levels comparable to those of the Casp6WT zymogen (Fig. 2b,c). FL, LSL, LS, and SS of Casp6 were confirmed with western blotting against anti-FL and anti-LSL Casp6 antiserum (Fig. 2d,e), neoepitope antiserum recognizing Casp6 cleaved at D179 (Fig. 2f,g), and anti-SS antibodies (Fig. 2h,i). No difference in the levels of each subunit between Casp6WT and Casp6N73T was detected (Fig. 2e,g,i). These results indicate that Casp6N73T zymogen self-processing is similar to that of Casp6WT zymogen.

### Recombinant Casp6N73T showed comparable VEIDase activity as Casp6WT on the tetrapeptide Ac-VEID-AFC substrate

**Figure 3:**
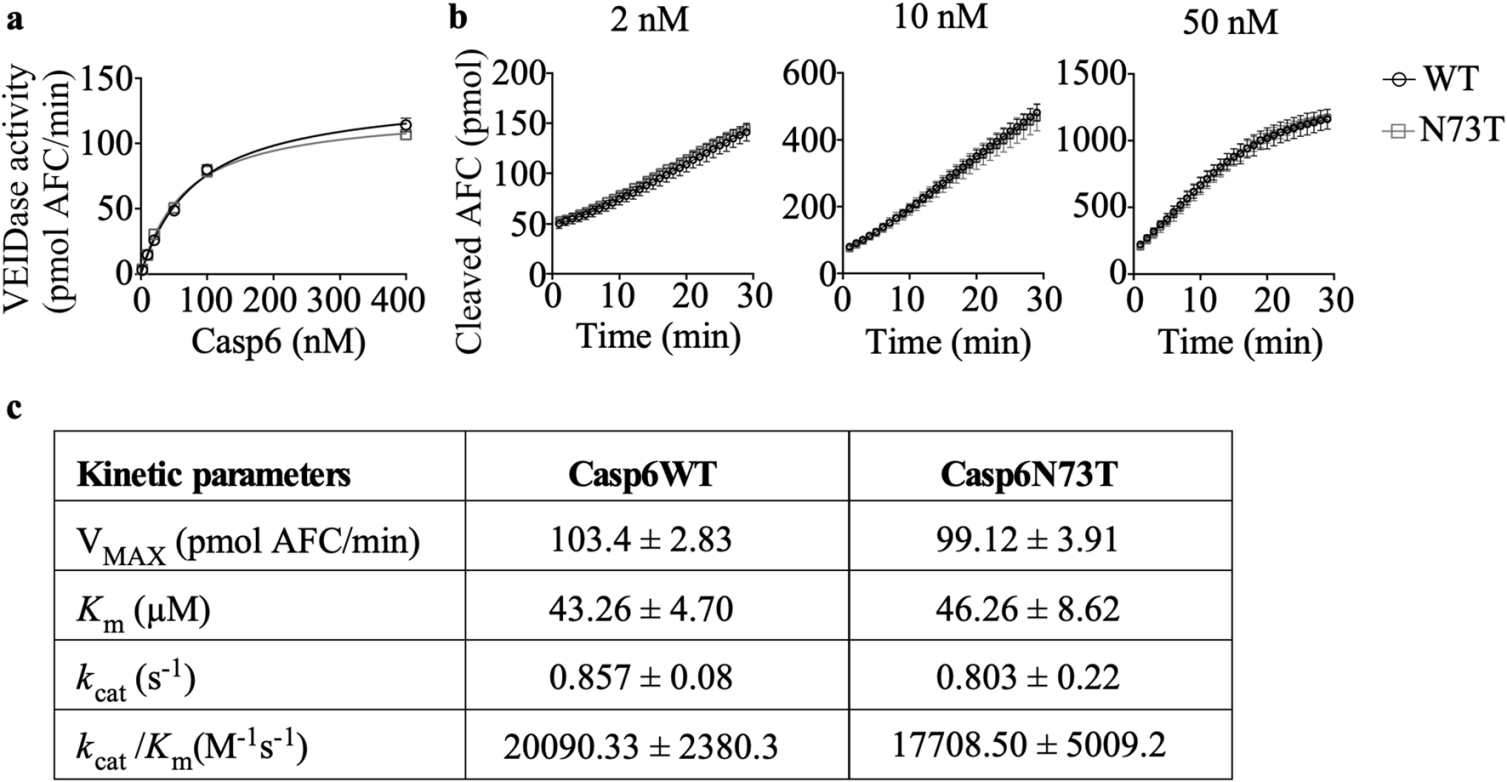
Casp6WT and Casp6N73T enzymatic processing of Ac-VEID-AFC. (**a**) VEIDase activity of Casp6WT or Casp6N73T at 2, 10, 20, 50, 100, or 400 nM on 20 µM Ac-VEID-AFC. (**b**) Cleaved Ac-VEID-AFC generated with time by Casp6WT or Casp6N73T at 2, 10, or 50 nM on 20 µM Ac-VEID-AFC. Data represents mean and s.e.m. from 3 independent experiments. No statistical differences were found by two-way ANOVA (**a**) or repeated measures two-way ANOVA (**b**). (**c)** V_MAX_, K_m_, k_cat_, and k_cat_/K_m_ values for Casp6WT and Casp6N73T. Data represents mean and SD from 3 independent experiments.

To determine whether Casp6N73T affects Casp6 catalysis, the catalytic efficiency of Casp6N73T on fluorescent tetrapeptide Ac-VEID-AFC was compared to Casp6WT. Before the activity assay, the number of active sites was established for each enzyme preparation by active site titration against de-esterified irreversible pan-caspase inhibitor, zVAD-FMK, to ensure comparison of equal amounts of Casp6WT and Casp6N73T active sites (Supplementary Fig. S1a-f). Two to four hundred nM active Casp6N73T exhibited comparable VEIDase activity (defined as cleaved pmol AFC per minute) than similar concentrations of active Casp6WT (Fig. 3a). The velocities of product formation by 2, 10, and 50 nM Casp6N73T with 20 µM Ac-VEID-AFC substrate were similar to those of 2, 10, or 50 nM Casp6WT (Fig. 3b). Furthermore, to better understand the kinetics of Casp6N73T, the K_m_, V_max_, k_cat_ and k_cat_/K_m_ were determined based on Michaelis–Menten kinetics measurements (Supplementary Fig. S2). No differences were found in the K_m_, V_max_, k_cat_ or k_cat_/K_m_ between Casp6N73T and Casp6WT (Fig. 3c).

### Recombinant Casp6N73T showed increased proteolytic processing of natural Casp6 protein substrate, Lamin A/C

**Figure 4:**
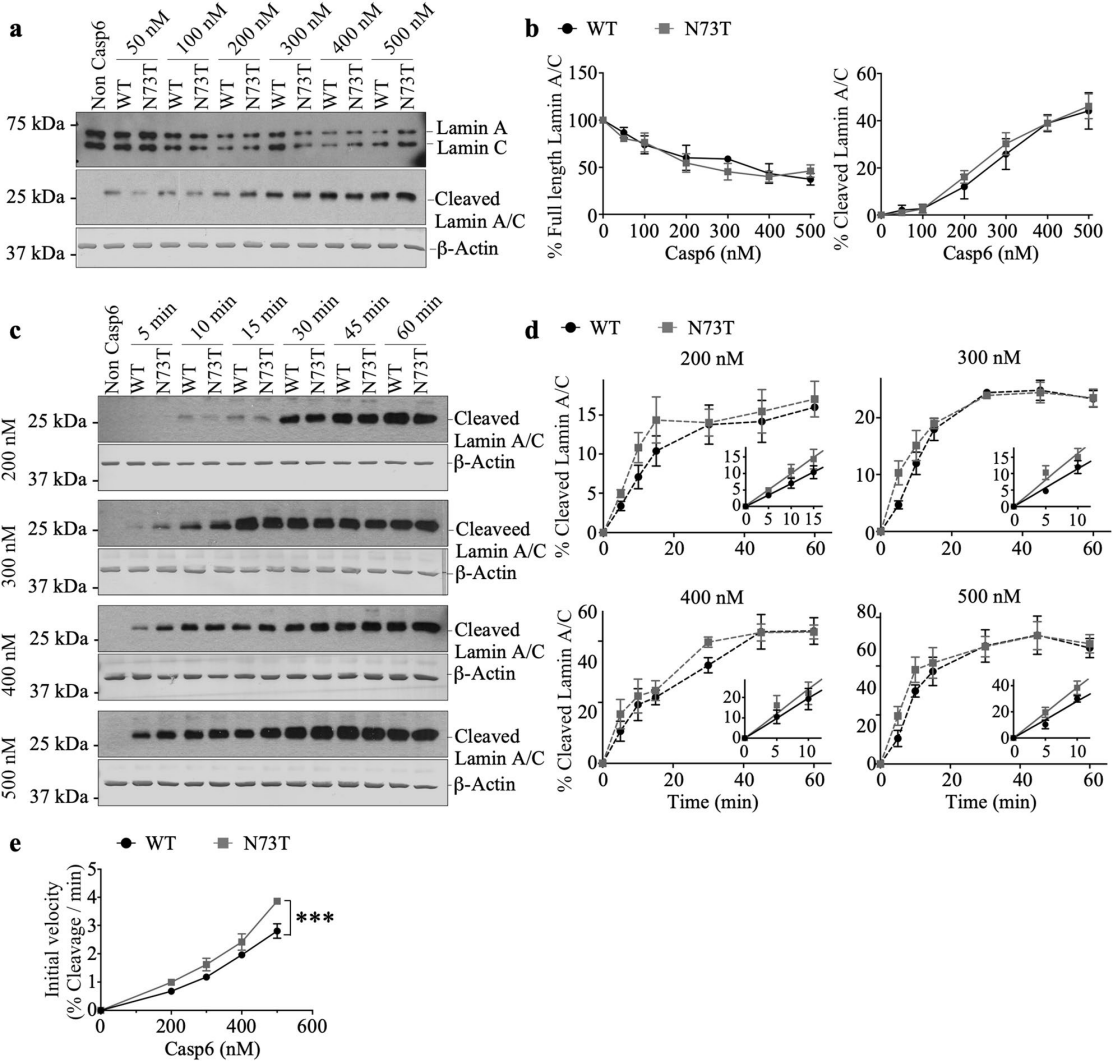
Casp6WT and Casp6N73T enzymatic processing of Lamin A/C. (**a**) Western blot of full-length (#2032 Cell Signaling Technology; top) or cleaved Lamin A/C at VEID (neoepitope antibody #2035 Cell Signaling Technology; middle) and β-Actin (bottom) from 50–500 nM recombinant Casp6WT or Casp6N73T incubated with Casp6 knockout mouse tissue nuclear extracts. (**b**) Quantification of full-length Lamin A/C and cleaved Lamin A/C by Casp6WT or Casp6N73T expressed as % of the control with no added recombinant active Casp6. (**c**) Western blots of time-dependent cleaved Lamin A/C by 200, 300, 400, or 500 nM Casp6WT or Casp6N73T incubated with Casp6 knockout tissue extracts. (**d**) Quantification of time-dependent generated cleaved Lamin A/C by Casp6WT or Casp6N73T shown in (**c**) expressed as % of the control with no added recombinant active Casp6. Insets: The initial velocity phase of the cleavage curve. The linear regression was performed on % cleaved Lamin A/C at 5, 10, and 15 min by 200 nM Casp6 (WT: R^2^ = 0.82, N73T: R^2^ = 0.82), or on % cleaved Lamin A/C at 5 and 10 min by Casp6 with 300 nM (WT: R^2^ = 0.88, N73T: R^2^ = 0.80), 400 nM (WT: R^2^ = 0.71, N73T: R^2^ = 0.66), or 500 nM (WT: R^2^ = 0.90, N73T: R^2^ = 0.90). (**e**) The initial velocity of Casp6WT and Casp6N73T reacting on Lamin A/C measured from (**d**). Data were shown as mean ± s.e.m from 3 independent experiments. Statistical evaluations were done with two-way ANOVA (variant: p = 0.0001, concentration: p < 0.0001, interaction: p = 0.1329). Post-hoc analyses were done with Tukey’s test. ***p < 0.001 N73T vs WT. Full-length images of blots/gels are presented in Supplementary Information.

To assess if Casp6N73T affects Casp6 activity on natural protein substrates, the cleavage of Lamin A/C by Casp6N73T and Casp6WT was assessed. Lamin A/C was extracted from Casp6 KO mouse tissue, and was incubated with varying concentrations of active site titrated Casp6N73T or Casp6WT. Both Casp6N73T and Casp6WT processed Lamin A/C and generated the expected 28 kDa fragment (Fig. 4a)^47^. The % FL Lamin A/C and cleaved Lamin A/C generated by Casp6N73T at concentrations from 50 to 500 nM were similar to that of Casp6 WT after 1 h incubation (Fig. 4b). In order to calculate the initial reaction velocity, cleaved Lamin A/C was measured every 5 min for the first 15 min of reaction, followed by every 15 min until 60 min. The amount of cleaved Lamin A/C increased with incubation time with both Casp6N73T and Casp6WT (Fig. 4c). Initially, at the 5 and 10 min time points, 200–500 nM Casp6N73T consistently generated slightly higher amounts of cleaved Lamin A/C than Casp6WT (Fig. 4d). The slope of the linear phase of the curve in Fig. 4d determined the initial velocity of the enzymes on Lamin A/C. The initial velocity of Casp6N73T was significantly higher than that of Casp6WT (Fig. 4e). These results suggest that the N73T substitution may modify Casp6N73T interaction with Lamin A/C.

### Recombinant Casp6N73T showed decreased catalytic efficiency on α-Tubulin

**Figure 5:**
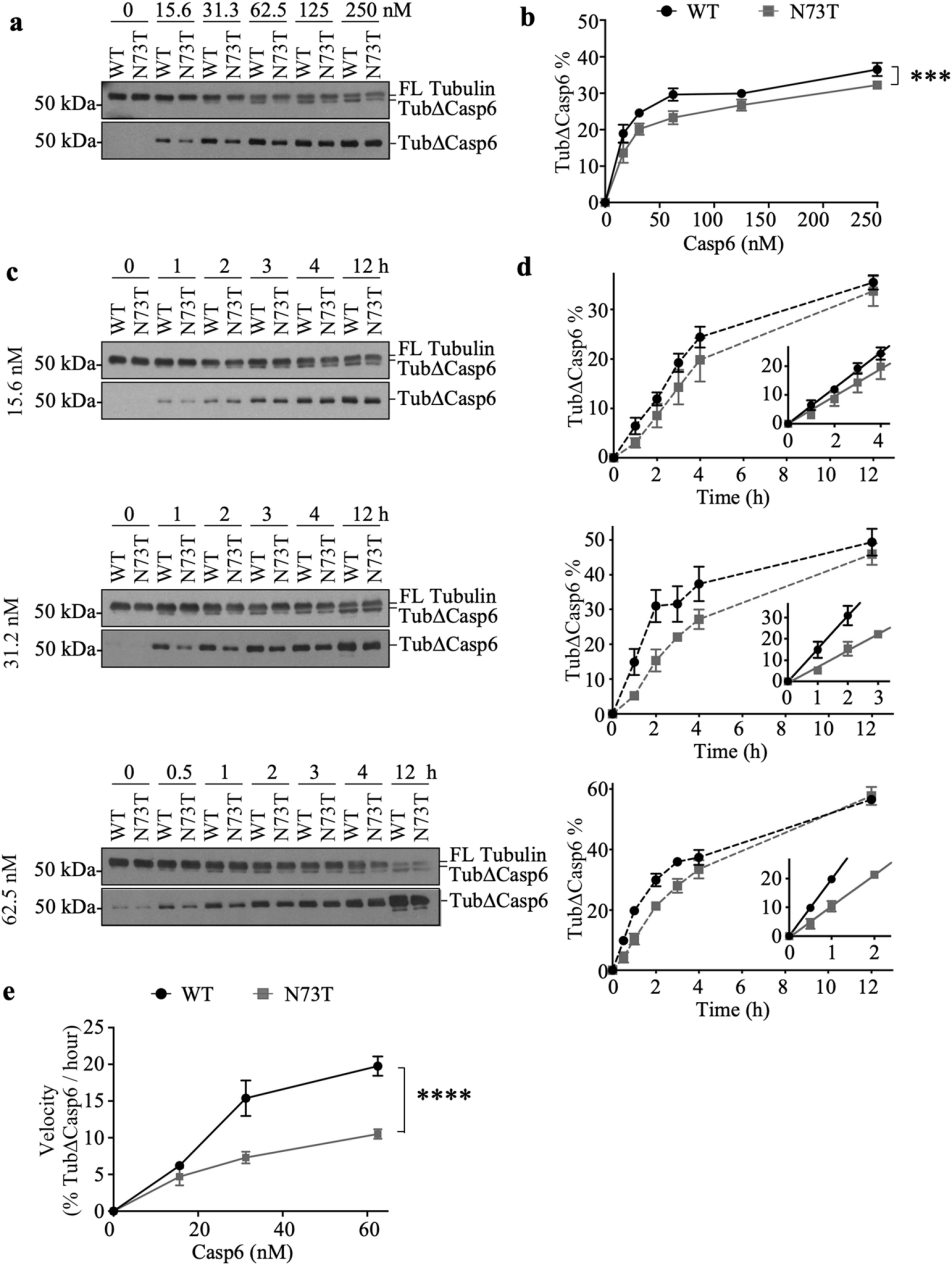
Casp6WT and Casp6N73T enzymatic processing of α-Tubulin. (**a**) Western blot of full-length (11H10 Cell Signaling Technology; top) or cleaved α-Tubulin at VGVD438 (GN20622; bottom) in the reactions of 15.6–250 nM recombinant Casp6WT or Casp6N73T with 242 nM of purified α- and β-Tubulin (121 nM of α-Tubulin). Tub∆Casp6, α-Tubulin cleaved by Casp6. (**b**) Quantification of Tub∆Casp6 shown in (A) expressed as % of FL Tubulin. Statistical evaluations were done with two-way ANOVA (variant: p = 0.0002, concentration: p < 0.0001, interaction: p = 0.4555). Post-hoc analyses were done with Tukey’s test. ***p < 0.001 N73T vs WT. (**c**) Western blot of time-dependent cleaved 121 nM α-Tubulin by 15.6, 31.2, or 62.5 nM Casp6WT or Casp6N73T. (**d**) Quantification of Tub∆Casp6 shown in (**c**) expressed as % of FL Tubulin. Insets: The initial velocity phase of the cleavage curve. The linear regression was performed on % Tub∆Casp6 at 1, 2, 3, and 4 h by 15.6 nM Casp6 (WT: R^2^ = 1.00, N73T: R^2^ = 0.99), on % Tub∆Casp6 at 1, 2, and 3 h by Casp6WT with 31.2 nM (R^2^ = 0.99) or 62.5 nM Casp6WT (R^2^ = 1.00), or on % Tub∆Casp6 at 1 and 2 h by Casp6N73T with 31.2 nM (R^2^ = 0.99) or 62.5 nM (R^2^ = 1.00). **(e)** Initial velocity of Casp6WT and Casp6N73T reacting on α-Tubulin measured from (**d**). Statistical evaluations were done with two-way ANOVA (variant: p < 0.0001, concentration: p < 0.0001, interaction: p = 0.0015). Post-hoc analyses were done with Tukey’s test. ****p < 0.0001 N73T vs WT. Data represents mean ± s.e.m. from 3 independent experiments. Full-length images of blots/gels are presented in Supplementary Information.

To further understand the effect of Casp6N73T on different protein substrates, the catalytic activity of Casp6N73T and Casp6WT on α-Tubulin was compared. Both Casp6N73T and Casp6WT processed α-Tubulin and generated a fragment 2 kDa smaller than the FL (Fig. 5a top panel), consistent with Casp6 cleavage at VGVD438 in α -Tubulin. Cleavage of α-tubulin at D438 was confirmed with the neoepitope antibody GN20622 (Fig. 5a bottom panel). The % Tub∆Casp6 by Casp6N73T at concentration 15.6–250 nM was significantly lower than that of Casp6WT after 4 h incubation (Fig. 5b). Kinetically, 15.6–62.5 nM Casp6N73T consistently generated less Tub∆Casp6 compared to Casp6WT during the first 4 h of incubation (Fig. 5c&d). Analysis of the initial velocity determined by the slope of the linear phase of the cleavage curve in Fig. 5d, showed a significant 50% reduction of Casp6N73T catalytic efficiency (**Fig. ****5****e**). These results show that Casp6N73T catalytic efficiency on α-Tubulin is lower than that of Casp6WT. As observed with the Lamin A/C substrate, the results suggest that Casp6N73T may interact differently than Casp6WT with α-Tubulin.

### The steady state level of active Casp6N73T subunits is considerably lower than those of Casp6WT in transfected human embryonic kidney 293 T (HEK293T) cells

**Figure 6:**
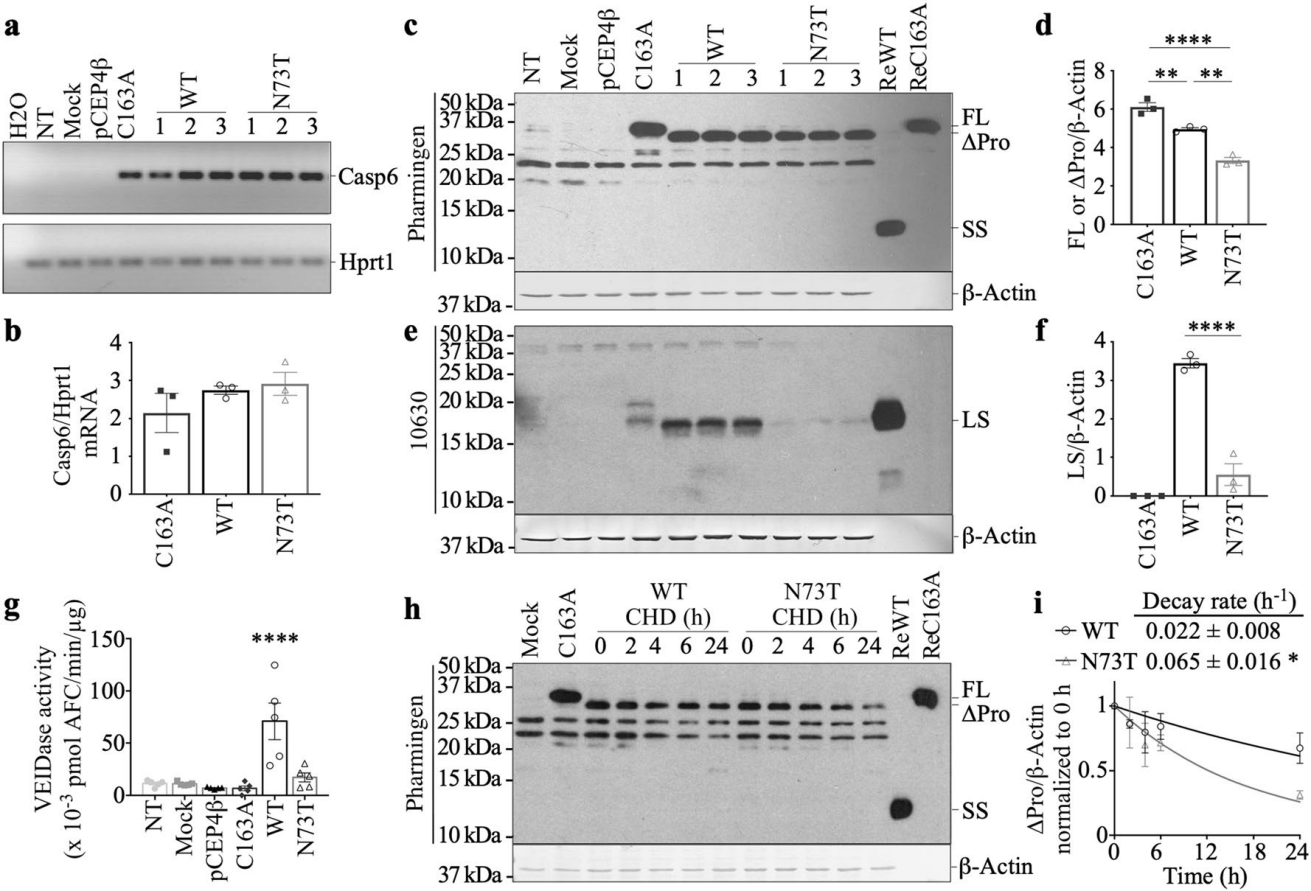
Steady levels of Casp6N73T and Casp6WT proteins in transfected HEK293T cells. (**a**) RedSafe-stained agarose gel of human Casp6 and Hprt1 mRNA amplicons in non-transfected (NT), mock, pCEP4β, pCEP4β-CASP6C163A-His, pCEP4β-CASP6WT∆Pro-His, or pCEP4β-CASP6N73T∆Pro-His-transfected cells. (**b**) Quantification of Casp6 mRNA levels normalized to Hprt1 shown in (**a**). (**c**,**e**) Western blot and (**d**,**f**) quantification of Casp6 in transfected cells against Pharmingen (**c**,**d**) or 10630 neoepitope antibodies (**e**,**f**). *ReWT* recombinant Casp6WT, *ReC163A* recombinant Casp6C163A, *FL* full length, *∆Pro* Casp6 without pro-domain, *SS* small subunit, *LS* large subunit. Statistical evaluations were done with one-way ANOVA (**d**,**f**: p < 0.0001) followed by post-hoc Tukey’s test (**p < 0.01, ****p < 0.0001). (**g**) VEIDase activity in protein extract from transfected cells. Statistical evaluations were done with one-way ANOVA (p < 0.0001) followed by post-hoc Tukey’s test. ****p < 0.0001 WT vs NT. (**h**) Western blot and (**i**) quantification of Casp6 in transfected cells with the Pharmingen antibody. The levels of ∆ProCasp6 normalized to the 0 h were fit to a one-phase decay to calculate the decay rate of Casp6WT or Casp6N73T. * indicates p = 0.012 done with unpaired t-test. Data shown represents mean ± s.e.m. from 3 independent experiments. Full-length images of blots/gels are presented in Supplementary Information.

To assess if eukaryotically expressed Casp6N73T behaves like the prokaryotically expressed recombinant Casp6N73T, *CASP6WT* and *CASP6N73T* cDNAs lacking their pro-domain (∆Pro) to promote self-processing of Casp6 were cloned in pCep4β and the constructs were transfected in human embryonic kidney 293 T (HEK293T) cells. Catalytically inactive, CASP6C163A with its pro-domain sequence, was transfected as a control for full length Casp6. Casp6 mRNA levels were equivalent in *CASP6WT, CASP6N73T, or CASP6C163A* cDNA transfected cells (Fig. 6a,b). In transfected cells, the ∆ProCasp6 expressed in *CASP6WT-* and *CASP6N73T*-transfected cells migrated approximately 2 kDa below the full length Casp6 in *CASP6C163A*-transfected cells (Fig. 6c). Unexpectedly, *CASP6N73T*-transfected HEK293T cells contained consistently lower levels of ∆Pro-Casp6 than *CASP6WT*-transfected cells (Fig. 6c,d). Compared to *CASP6C163A*-transfected cells, ∆ProCasp6 levels were lower in *CASP6WT-*transfected cells, as expected since Casp6WT can be processed into its subunits (Fig. 6d). The levels of LS in *CASP6N73T-*transfected cells were significantly lower than those of *CASP6WT-*transfected cells (Fig. 6e,f). Consistently, *CASP6N73T-*transfected HEK293T protein extracts did not show significant Casp6 VEIDase activity compared to non-, mock-, or empty vector-transfected cell extracts, while *CASP6WT*-transfected cells exhibited high VEIDase activity (Fig. 6g). Because transfected cells contained similar Casp6WT and Casp6N73T mRNA levels, decreased Casp6N73T full length protein level and VEIDase activity may be the result by enhanced turnover. Indeed, in the presence of translational inhibitor cycloheximide (CHD), the degradation rate of ∆ProCasp6N73T was faster than that of ∆ProCasp6WT levels (Fig. 6h,i), suggesting a higher cellular turnover of Casp6 due to the N73T substitution. Since Casp6 LS has previously been reported to be degraded by the proteasome^13,48^, we verified if proteasomal activity may be responsible for FL or LS Casp6N73T lower levels. The epoxomicin proteasomal inhibitor did not significantly alter levels of either Casp6N73T FL or LS in HEK293T cells, excluding proteasomal degradation as an explanation (Supplementary Fig. S3). Nevertheless, these results indicate that Casp6N73T is unstable and degraded by an alternate non-proteasomal dependent mechanism, thereby limiting the amount of Casp6N73T LS and Casp6N73T activity produced in mammalian cells.

### Recombinant Casp6N73T caused less neurofunctional damage and neuronal degeneration than Casp6WT in hippocampal CA1 pyramidal neurons

**Figure 7:**
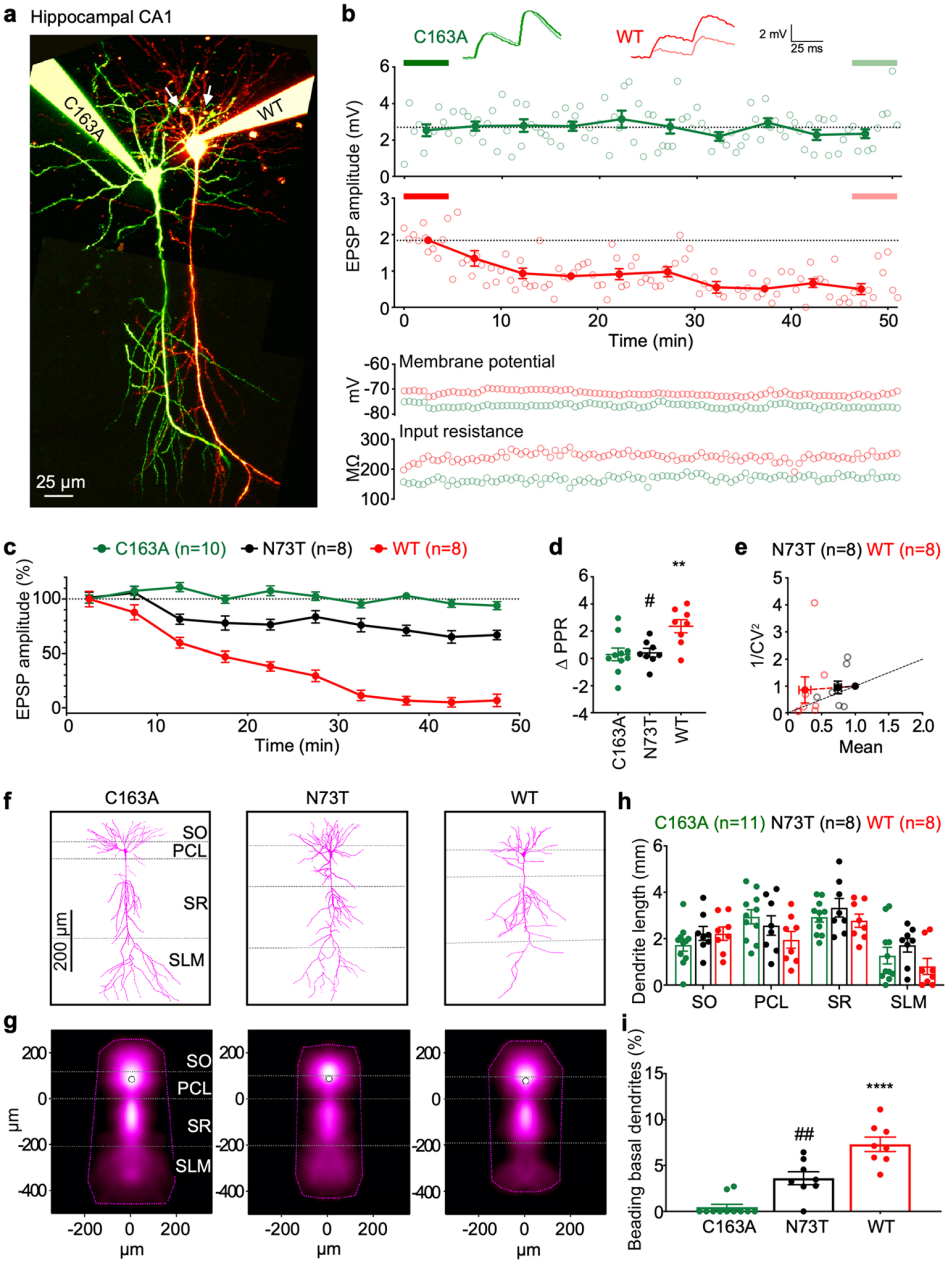
Casp6N73T is less damaging to neuronal function and neurodegeneration than Casp6WT. (**a**) Representative two-photon images of 10 pg Casp6C163A- (Alexa 488, green) and 10 pg Casp6WT- or Casp6N73T- (Alexa 594, red) patched hippocampal CA1 pyramidal neurons. White arrows indicate basal dendrite degeneration. Scale bar: 25 µm. Maximum-intensity projection of two-photon stacks was compiled using ImageJ, and imaging montage of entire neurons was performed using Affinity Designer 1.7. (**b**) Sample EPSP time course plots from Casp6C163A- (green) and Casp6WT- (red) patched neurons in (**a**), showing reduction of neurotransmission for Casp6WT (1.53 ± 0.32 mV, n = 8 vs. 0.14 ± 0.07 mV, n = 8, p < 0.01) but not for Casp6C163A (1.94 ± 0.32 mV, n = 10 vs. 1.91 ± 0.29 mV, n = 10, p = 0.76) when comparing the last 10 traces (light thick line) to the first 10 traces (dark thick line). Open circles: EPSP amplitude recorded every 30 s. Closed circles: EPSP amplitude binned and averaged across 10 traces. Inset: representative EPSP traces highlight the paired-pulse ratio (PPR). Scale bars: 2 mV, 25 ms. Resting membrane potential and input resistance remained stable throughout experiment. (**c**) EPSP time courses for Casp6C163A- (n = 10), Casp6N73T- (n = 8), or Casp6WT- (n = 8) patched neurons. (**d**) PPR from Casp6WT-, Casp6C163A- or Casp6N73T-patched neurons. One-way ANOVA (p = 0.0043), followed by Tukey’s post-hoc test (**p < 0.01 vs. C163A; #p < 0.05 vs. WT). (**e**) CV analysis of Casp6N73T- and Casp6WT-patched neurons. Casp6C163A was unaltered. (**f**) Representative reconstructions of Casp6C163A-, Casp6N73T-, or Casp6WT-patched neurons. Image stacks were used for manual reconstruction of 3D morphologies using the Neuromantic freeware (http://www.reading.ac.uk/neuromantic/body_index.php). (**g**) Dendritic density maps of Casp6C163A- (n = 11), Casp6N73T- (n = 8), or Casp6WT- (n = 8) patched neurons generated using custom software running in Igor Pro 8 v8.04 (https://www.wavemetrics.com, https://github.com/pj-sjostrom/qMorph). Dotted lines show the convex hull of the maximum extent. (**h**) Cumulative dendritic length of reconstructed neurons in layers SO, PCL, SR, and SLM. (**i**) Casp6C163A-, Casp6N73T- and Casp6WT-patched CA1 pyramidal neurons beading basal dendrites. One-way ANOVA (p = 0.0001), followed by Tukey’s test (****p < 0.0001 vs Casp6C163A, ##p < 0.01 vs Casp6WT).

The effect of Casp6N73T on neuronal function was compared with recombinant Casp6WT and catalytically inactive Casp6C163A proteins by patching CA1 pyramidal neurons in hippocampal organ slices with these proteins (Fig. 7a) and measuring the amplitude of excitatory postsynaptic potential (EPSP) (Fig. 7b). EPSP amplitudes from recorded neurons were analyzed when the membrane potential and input resistance were stable (Fig. 7b bottom panel). Activated Casp6WT and Casp6N73T were stable for 5 h after their preparation in internal solution (Supplementary Fig. S4), within which the recording data was acquired. Ten pg Casp6WT induced a decrease of EPSP amplitude 30 min after patching, and a continually decreasing EPSP amplitude during the 50 min of recording (Fig. 7c). In contrast 10 pg catalytically inactive Casp6C163A did not alter EPSP amplitude within 50 min. Interestingly, 10 pg Casp6N73T decreased the EPSP amplitude less than Casp6WT in pyramidal neurons for approximately 10 min after patching, and EPSP amplitude was maintained at 65% of the original levels for 50 min (Fig. 7c). Paired pulse ratio (∆PPR) of the first and second EPSP wave increased in Casp6WT patched neurons compared to Casp6C163A, suggesting that the decaying EPSP amplitude was at least in part due to a reduced probability of release presynaptically^49^.

In contrast, Casp6N73T ∆PPR was indistinguishable from that of Casp6C163A (Fig. 7d), possibly hinting at an absence of perceivable effects on presynaptic release. Although the change in PPR due to Casp6WT suggested a presynaptic locus of action, the coefficient of variation (CV) analysis of the first EPSP^50^ indicated a predominantly postsynaptic effect, although with variable outcome (Fig. 7e). Taken together, the combined outcome of PPR and CV analyses is consistent with a coordinated downregulation on both pre- and post-synaptic sides due to Casp6WT.

The morphology of Casp6WT, Casp6N73T, or Casp6C163A patched CA1 pyramidal neurons was analyzed by manual 3D reconstruction (Fig. 7f). Dendritic map density and convex hulls indicated the average distribution and the maximum extent of dendrites, respectively (Fig. 7g). Neurons patched with Casp6WT exhibited a lower length of apical dendrites in the SLM region compared to Casp6C163A-patched neurons, but the data did not reach statistical significance (Fig. 7h). The apical dendrites length of Casp6N73T-patched neurons was longer than those of Casp6WT-patched neurons. In addition, Casp6WT-patched neurons displayed beading along basal neurites in the stratum oriens region indicative of neuronal degeneration (Fig. 7i), while beading was rarely seen in neurons patched with Casp6C163A, indicating that this beading is linked to Casp6 activity. The beading dendrites of Casp6N73T-patched neurons was approximately 50% lower than in neurons patched with Casp6WT. These results indicate that Casp6N73T is less detrimental to neuron function and causes less neurodegeneration than Casp6WT.

## Discussion

Our study demonstrates that a rare human *CASP6* variant encoding Casp6N73T, genetically associated with preserved hippocampal CA1 volume in an AD cohort, exhibits altered catalyses on natural protein substrates, lamin A and α-Tubulin. In addition, our study showed that Casp6N73T has less negative impact on neuronal function and neurodegeneration than the Casp6WT in mice CA1 hippocampal pyramidal neurons. These results provide initial evidence for a neuroprotective effect by Casp6N73T as reflected by hippocampal subfield volume preservation but this needs to be validated in independent data sets.

The significant association between Casp6N73T and hippocampal CA1 volume by SKAT-O provides an advantage in identifying associations between low frequency functional exonic SNPs and pathological features of AD compared to genome-wide association studies (GWAS)^45,51^. Different from the single regression model used in GWASs, SKAT uses a multiple regression model to regress the phenotypes of different SNPs in the same genomic region and allows different orientations of the phenotype, in order to assess the cumulative effects of one gene on disease. Another advantage of our approach is the use of a quantitative measure of hippocampal CA1 volume, instead of the numerous variables of clinically diagnosed AD. Hippocampal CA1 volume was chosen because atrophy in this region occurs early in AD progression^52,53^. Large scale longitudinal measures indicate that hippocampal CA1 atrophy is a robust MRI biomarker to distinguish MCI that further develop to AD from stable MCI^54^. Furthermore, rare SNPs also mean lower number of individuals carrying the SNP, therefore, our finding will require testing additional cases to confirm the role of Casp6N73T in MCI and AD. On the other hand, the ADNI study provides precise cognitive and neuroimaging information on the individual carrying *CASP6*N73T. The Casp6N73T variant was identified in a heterozygous individual at the early MCI stage with unexpected preserved hippocampal CA1 volume compared to those carrying the wild type *CASP6*.

Here, Casp6N73T was shown to be less damaging to synaptic transmission and plasticity than Casp6WT. In contrast to catalytically inactive Casp6C163A, Casp6WT rapidly decreased induced EPSP amplitude in CA1 hippocampal neurons, whereas Casp6N73T showed only a minor decrease. In addition, the paired pulse ratio was increased in Casp6WT, but not in Casp6N73T. Furthermore, Casp6WT, but not Casp6C163A, induced basal dendritic beading indicating Casp6 activity-mediated neurodegeneration CA1 neurons, a feature that was reduced with Casp6N73T. Based on the morphological analysis of reconstructed neurons, Casp6WT, but not Casp6N73T, caused a decrease in the total apical dendritic length. These data are consistent with transgenic expression of a self-activated form of Casp6WT in CA1 neurons causing age-dependent cognitive impairment^11^, small amounts of active Casp6 causing a rapid depression of CA1 neuronal transmission^22^ and impairing long-term potentiation in hippocampal CA1 circuits in vivo^55^. Given that the presence of active Casp6 detected in the entorhinal cortex of aged individuals correlates with decreased cognitive performance^1,7,12,42^ and that early cognitive decline is strongly correlated with synaptic loss^56,57^, these data raise the possibility that the active Casp6 observed in neuropil threads, neuritic plaques and neurofibrillary tangles^4,5^ causes synaptic transmission problems. Most importantly, the data suggest that Casp6N73T variant would not be as detrimental as Casp6WT for neuronal structure and function and this may protect aged individuals from cognitive decline. In support of this conclusion, Casp6 deficient mice have been shown to be protected against excitotoxicity, nerve growth factor deprivation and myelin-induced axonal degeneration, and showed an age-dependent increase in cortical and striatal volume^58^.

The molecular reason for the protective action of Casp6N73T might stem from its altered proteolysis of natural protein substrates compared to Casp6WT. Casp6 can cleave numerous protein substrates, some of which are specific to neurons and synapses^2,59^ and involved in neurodegenerative diseases, such as Tau, Vimentin, Drebrin, Spinophilin, α-Actinin^2,5^, valosin containing protein p97^3^, APP^60^, Presenilin 1 and 2^28^, and Huntingtin^61^, that may be responsible for structural damage, synaptic loss, and protein aggregation. Here, we show that compared to Casp6WT, Casp6N73T had increased proteolytic processing of Lamin A/C, decreased proteolysis of α-tubulin and equivalent processing of the Ac-VEID-AFC peptide substrate. These results indicate that either the N73T alters the structure of the Casp6 enzyme thereby affecting the catalytic site, or the N73T alters the interaction of Casp6 with substrates. The altered catalysis of natural protein substrates α-Tubulin and Lamin A/C, but not of the small peptide substrate, suggests that the active site of the Casp6N73T is relatively normal. Therefore, it is likely that Casp6N73T interacts differently with α-Tubulin and Lamin A/C than Casp6WT, thus suggesting the presence of an exosite either within N73, its surroundings, or in a structure that is altered by N73T. Exosites are defined as sites outside the substrate-binding catalytic site which influence structure or function of an enzyme. The N73T substitution is located in the middle of Casp6’s helix B with a shorter side chain pointing outward. The nearby negatively charged D72 is conserved among Casp6 orthologues and unique to Casp6 among the caspase protein family, which may also contribute to this exosite. Casp6 exosite 42RRR44, located at the hinge between the core structure and the N-terminus of the large subunit, has been confirmed^62,63^. Furthermore, two rare human variants, G66R and R65W completely eliminate or significantly reduce Casp6 activity through impaired substrate binding, alter the catalytic site activity, and have dominant negative effects on Casp6 WT^46^. The alternatively spliced Casp6b isoform lacking amino acids 13–104 while retaining the catalytic site also has no activity, and inhibits Casp6WT activation^64^. The N73T data presented here further highlights the importance of the N-terminus of the large subunit of Casp6 in substrate recognition. Casp6N73T may change the proteolysis of different neuronal substrates in addition to α-Tubulin, which together display a reduced damage due to Casp6 and favors a neuroprotective phenotype. Future investigation in characterizing the cleavage efficiencies of Casp6N73T on these substrates could expand our understanding of the new exosite as well as the protective mechanism of Casp6N73T. The identification of an additional exosite for Casp6 could be useful in specific drug design against Casp6 activity, which has been shown to cause age-dependent cognitive impairment in mice^11^.

The protective action of Casp6N73T might also stem from its instability in mammalian cells. Casp6N73T full length levels were decreased significantly relative to Casp6WT, despite similar mRNA levels for Casp6N73T and Casp6WT. This lower level of full length Casp6N73T could be explained by either a lower mRNA translational rate, increased protein processing, or increased degradation by alternate cellular proteolytic activities. A more rapid turnover for full length Casp6N73T compared to FL Casp6WT was observed indicating either increased protein processing into its active subunits or increased degradation by alternate cellular proteolytic activities. The fact that the level of Casp6N73T processed large subunit (LS) was reduced almost tenfold relative to Casp6WT indicated that Casp6N73T was processed less efficiently than Casp6WT or that the Casp6N73T LS was degraded more rapidly than the Casp6WT LS. Proteasomal activity was excluded since proteasomal inhibition did not significantly alter either FL or LS Casp6N73T levels. The fact that self-processing of prokaryotically expressed recombinant Casp6N73T is equivalent to that of Casp6WT eliminates the possibility that the Casp6N73T mutation alters processing. Therefore, the most likely explanation for these findings is that the FL Casp6N73T is unstable and degraded by a non-proteasomal cellular mechanism, thereby limiting the amount of Casp6N73T LS and Casp6N73T activity produced in mammalian cells.

Lastly, we confirm here that *CASP1* and *CASP6* mRNA levels are significantly increased in specific regions of the AD brains in the ADNI cohort compared to cognitively normal control brains from large sample sizes in the AMP-AD Consortium. We investigated *CASP1* expression because it activates Casp6 in human primary neurons^42^. Increased *CASP1* mRNA levels have been reported previously in AD cortex and entorhinal cortex (ERC)^65,66^. Our results additionally show increased *CASP1* mRNA levels in AD temporal cortex and superior temporal gyrus. *CASP6* expression in AD is more controversial. One early study reports low level of *CASP6* mRNAs in AD brains^65^. Others indicate increased *CASP6* mRNA levels in cortex and cerebellum of AD brains^43,66^. Here, we show increased *CASP6* mRNA levels in AD temporal cortex, superior temporal gyrus, para-hippocampal gyrus, and dorsolateral prefrontal cortex. These results support the implication of *CASP6* in AD.

This study highlights the importance of assessing the role of the amazing genetic diversity of humans in disease by combining human genetic information associated with well-ascertained neuroimaging and cognitive measures with biochemical and electrophysiological approaches to investigate the potential influence of rare variants on AD-related pathologies and cognition.

## Methods

### Alzheimer’s Disease Neuroimaging Initiative (ADNI)

Individuals used in the analysis were ADNI participants o^67,68^. Inclusion and exclusion criteria, clinical and neuroimaging protocols, and other information about ADNI can be found at www.adni-info.org and http://www.loni.usc.edu/ADNI/. Written informed consent was obtained at the time of enrollment for imaging and genetic sample collection and protocols of consent forms were approved by each participating sites’ Institutional Review Board. Human subject ethical approval was obtained by ADNI and can be found at http://www.loni.usc.edu/ADNI/. All methods and experiments were performed in accordance with relevant guidelines.

### Whole genome sequencing (WGS) analysis

WGS on the Illumina HiSeq2000 platform with paired-end reads was performed on blood-derived genomic DNA samples obtained from 817 ADNI participants^69^. Briefly, short-read sequences were mapped to the human genome assembly (GRCh build 37.72) using BWA^70^. During the alignment, we use only bases with Phred Quality > 15 in each read to include soft clipping of low-quality bases, retain only uniquely mapped pair-end reads, and remove potential PCR duplicates. After completing initial alignment, the alignment was further refined by locally realigning any suspicious reads. The reported base calling quality scores obtained from the sequencer were re-calibrated to account for covariates of base errors. All variants with statistical evidence for an alternate allele present among individuals were identified using GATK HaplotypeCaller for multi-sample variant callings.

### Neuroimaging analysis

Baseline T1-weighted brain MRI scans were downloaded from the ADNI database. FreeSurfer software was used to process T1-weighted brain MRI scans^71^ and extract region of interest (ROI)-based imaging phenotypes^72,73^.

### Gene-based association analysis

Since population stratification is known to cause spurious association in disease studies, we restricted our analyses to only subjects that clustered with CEU (Utah residents with Northern and Western European ancestry from the CEPH collection) + TSI (Tuscany in Italy) populations using HapMap 3 genotype data and the multidimensional scaling analysis (www.hapmap.org)^74–76^. A total of 757 ADNI participants (259 CN, 219 early MCI, 232 late MCI, and 47 AD) were used for analysis, where late and early MCI were defined as the cognitive performance below 1.5 standard deviations of the normative mean on a standard test and at the range of 1 to 1.5 standard deviations, respectively^77^. After extracting WGS-identified functional exonic SNPs within *CASP6*, we performed a gene-based association analysis of rare variants (minor allele frequency < 0.05) using SKAT-O software^78^. For hippocampal CA1 volumes, age, sex, year of education, MRI field strength, and total intracranial volume were used as covariates.

### RNA-Seq analysis

RNA-Seq data (n = 1,966 individuals for the seven brain regions) reprocessed and realigned in the AMP-AD Consortium using a RNA-Seq pipeline^79^, were downloaded from the Sage Bionetworks (www.synapse.org). Human postmortem brain RNA-Seq data were obtained in three independent studies (Religious Orders Study and the Memory and Aging Project (ROS/MAP), Mount Sinai School of Medicine (MSSM), and Mayo Clinic) from seven distinct brain regions. Differential gene expression of *CASP6* and *CASP1* between AD and cognitively normal controls were analysed with R package limma^80^.

### Mutagenesis and sub-cloning of Casp6N73T

The pET23b( +)-Casp6WT-His plasmid (Addgene #11,823 https://www.addgene.org/11823/, gift from Dr. Guy Salvesen, Burnham Institute, La Jolla, CA, USA) encodes a human Casp6WT zymogen fused with a C-terminal 6 × His tag. The mammalian pCEP4β- Casp6∆ProWT-His plasmid encodes a human Casp6WT lacking its pro-domain^14^. Both plasmids were mutated using QuikChange Site-Directed Mutagenesis Kit (Stratagene, San Diego, CA, USA) with Casp6N73T primers 5'- GCG CAG ATA GAG ACA CTC TTA CCC GCA GG -3’ and 5'- CCT GCG GGT AAG AGT GTC TCT ATC TGC GC -3' to introduce the mutation for the N73T substitution. All constructs were verified by sequencing at McGill University Genome Quebec Innovation Center.

### Protein expression and purification

The pET23b( +)-Casp6WT-His or pET23b( +)-Casp6N73T-His plasmids were expressed in BL21(DE3)pLysS competent cells (Promega, Madison, WI, USA). Casp6 was purified using Ni Sepharose Fast Flow 6 (GE Healthcare, Chicago, IL, USA) and Macro Prep High Q Resin (Bio-Rad Laboratories, Hercules, CA, USA) as described^32,46^. Pure protein was aliquoted, fast frozen in EtOH/dry ice bath, and stored at − 80 °C freezer.

### Active site titration

Casp6 active site concentration was determined using an irreversible inhibitor N-benzyloxycarbonyl-Val-Ala-Asp-(O-methyl)-fluoromethylketone (zVAD-FMK; MP Biomedicals, Irvine, CA, USA) as described^46^.

### Caspase activity assays on Ac-VEID-AFC

The release of AFC from 20 µM Ac-VEID-AFC by active site titrated Casp6 (2, 10, 20, 50, 100, or 400 nM) was measured as described^46^. The slope of the linear phase of cleaved AFC plotted against time was calculated as VEIDase activity.

### *Determination of K*_*m*_* and k*_*cat*_

Release of AFC from 1–200 µM Ac-VEID-AFC by 20 nM Casp6 at 37 °C in Stennicke buffer was measured as described^46^. VEIDase activity versus Ac-VEID-AFC concentration was fitted to a Michaelis–Menten equation v = (V_max_ × [S])/(K_m_ + [S]) using Prism 7 software (GraphPad Software, CA, USA) and maximal velocity V_max_, Michaelis–Menten constant K_m_ , and k_cat_ = V_max_/[active site concentration of Casp6] were calculated.

### Casp6 activity assay on protein substrates

To assess Lamin A/C cleavage, nuclear proteins were extracted from Casp6 knockout mice colon tissue^81^ as described^46^. Casp6 (50–500 nM) were incubated with 3 µg lysate in Stennicke buffer at 37 °C for 5–60 min.

To assess α-Tubulin cleavage, purified porcine Tubulin (Cytoskeleton, Inc., Denver, CO, USA) was used. Tubulin powder was reconstituted in General Tubulin Buffer (80 mM PIPES pH 6.9, 2 mM MgCl_2_, 0.5 mM EGTA) supplemented with 10 mg/mL GTP. Casp6 (15.6–250 nM) was incubated with 252 nM Tubulin in Stennicke buffer at 37 °C for 1–12 h.

After the incubation period, samples were prepared in Laemmli buffer and analyzed by Western blot. The initial velocity of Casp6 on Lamin A/C or α-Tubulin was calculated based on the linear portion of the cleavage curve. The number of data points in the linear portion of each cleavage curve used for linear regression depended on the goodness-of-fit when R^2^ was closest to 1.00.

### Western blots

Fifty ng recombinant Casp6, 6 µg nuclear or 20 µg HEK293T protein extracts, 0.05–0.2 µg Tubulin, were submitted to western blot analyses with 1:1000 mouse anti-Casp6 against human Casp6 amino acids 24–293 (SC-81653, Santa Cruz Biotechnology, Dallas, TX, USA), 1:10,000 rabbit neoepitope 10630 antiserum^5^, or 1:250 mouse anti-Casp6 small subunit (BD Pharmingen clone B93-4, BD Biosciences, San Jose, CA, USA), 1:1000 anti-Lamin A/C (Cell Signaling Technology #2032, Danvers, MA, USA), 1;1000 anti-cleaved Lamin A/C at VEID^230^ (Cell Signaling Technology #2035), 1;1000 anti- α-Tubulin (Cell Signaling Technology #11H10), 1:5000 anti-cleaved α-Tubulin at VGVD^438^^14^, or 1:5000 anti-β-Actin ( Sigma-Aldrich Co # A5441, Oakville, ON, Canada). Immunoreactivity was detected with 1:5000 HRP-conjugated anti-rabbit (DAKO P0217, Agilent Technologies Burlington, ON, Canada) or anti-mouse (Jackson ImmunoResearch Labs #133499, West Grove, PA, USA) secondary antibody followed by ECL (GE Healthcare), or with alkaline phosphatase-conjugated anti-mouse secondary antibody (1:5000; Jackson) followed by NBT/BCIP (Promega). Immunoreactive bands were scanned, and densitometry was performed using ImageJ software (NIH, Bethesda, MD, USA) by measuring band intensity values beyond the background.

### Transfection, treatments, and protein extraction of HEK293T cells

The transfections were carried out with Lipofectamine 2000 (Invitrogen, Burlington, ON, Canada) and proteins extracted after 24 h in cell lysis buffer for Casp6 activity or in RIPA for western blots as described^14^. Transfection efficiency of Lipofectamine 2000 was optimized to obtain more than 90% transfected cells by calculating the number of fluorescent cells transfected with pBud-EGFP plasmid (Addgene #23027) over the number of total cells stained with Hoechst (Thermo Fisher Scientific). For protein degradation analysis, cells were treated 24 h after transfection with 75 µg/ml cycloheximide (CHD, Sigma-Aldrich) for the indicated times.

### Treatment of transfected HEK293T cells with epoxomicin

Cells were treated with 50 nM of epoxomicin (Enzo) 24 h after transfection for the indicated times and proteins extracted in cell lysis buffer. Proteasomal inhibition was confirmed in a proteasome activity assay as reported previously^48^ using 50 µM Succinyl-Leu-Leu-Val-Tyr-7-amino-4-methylcoumarin (Suc-LLVY-AMC; Enzo).

### RT-PCR

Total RNA was extracted using TRIzol (Invitrogen) and converted to cDNA with avian myeloblastosis reverse transcriptase (Roche Diagnostics, Laval, QC, Canada). Casp6 cDNA was amplified with Taq DNA polymerase (New England Biolabs, Ipswich, MA, USA) as described^46^.

### Electrophysiology

All animal experimentation was approved by the McGill University Animal Care Committee and performed under guidelines and regulations in accordance with the ARRIVE guidelines. Hippocampal slices (300 µm) from 11–16 days old C57BL/6 mice were prepared in ice-cold ACSF^22^.

Recombinant Casp6 were activated in Stennicke buffer at 37 °C for 15 min, and diluted to 10 pg/10 µL in internal solution^82^ with 30–60 µM Alexa 594 or 40–80 µM Alexa 488 (Invitrogen), filtered through 0.22 µm hydrophilic polyethersulfone filters (Millipore Sigma, Oakville, ON, Canada), and osmolarity double checked to be ~ 310 mOsm.

Multiple whole-cell patches were performed as described^82^. The stimulating electrode was placed in the CA1 stratum orients and delivered five 0.1-µs-long biphasic pulses (10–50 V) at 30 Hz every 30 s to elicit EPSPs in the range 1–3 mV. The recording was done for 1 h unless the data failed to meet the stability criteria defined as potential (± 4 mV), input resistance (± 15%) and temperature (32–34 °C). Offline data were analysed with Igor Pro (WaveMetrics Inc.). EPSP peak amplitudes were measured and averaged every 2.5 min (5 traces). Ensemble time courses were normalized to the first 2.5 min EPSP.

### Two-photon imaging and neuron reconstruction

Two-photon excitation was achieved using a Chameleon Ultra II femtosecond laser (Coherent, Santa Clara, CA, USA) tuned to 780 nm for both Alexa 594 and 488. Two-photon microscopes were custom-built^83^. Imaging data were acquired using customized versions of ScanImage 2018 (Vidrio Technologies, Leesburg VA USA)^84^ running in Matlab (The MathWorks, Natick, MA, USA) via a PCI-6110 or a PCIe-6374 data acquisition board (National Instruments, Austin, TX, USA). When neurons had been well loaded with dye (> 1 h after break-in), the neuronal morphology was acquired as stacks of 512-by-512-pixels slices at 2 ms/line spaced by 2 µm. Maximum-intensity two-photon-imaging stacks compiled with ImageJ (NIH) were used for morphological identification (Fig. 7a), and for quantification of basal dendrite beading. Imaging montage of entire neurons was performed by Affinity Designer 1.7 (Serif Ltd, West Bridgford, Notts, UK). Image stacks were used for manual reconstruction of 3D morphologies (Fig. 7f) using the Neuromantic freeware(http://www.reading.ac.uk/ neuromantic/body_index.php). Morphometry of 3D reconstructions (e.g. density maps, hulls, etc. in Fig. 7g,h) was subsequently performed using custom software^83^ running in Igor Pro 8 (WaveMetrics Inc.). CA1 layer boundaries were identified using laser-scanning Dodt contrast images acquired simultaneously with 2-photon fluorescence.

### Statistic study

Statistical analyses of data were performed using Igor Pro 8 or GraphPad Prism 7 with Student’s t-test or one/two-way ANOVA, as indicated in figure legends.

## Supplementary Information

Supplementary Information.

## Data Availability

Materials generated for this study are available upon request. The datasets supporting the conclusions of this article are available in ADNI database (http://www.loni.usc.edu/ADNI/) for whole genome sequencing and MRI scan data the Sage Bionetworks (www.synapse.org) for human postmortem brain RNA-Seq data from seven brain regions.
