# Author Correction: Rare *CASP6*N73T variant associated with hippocampal volume exhibits decreased proteolytic activity, synaptic transmission defect, and neurodegeneration

**DOI:** 10.1038/s41598-021-94736-x

**Published:** 2021-08-03

**Authors:** Libin Zhou, Kwangsik Nho, Maria G. Haddad, Nicole Cherepacha, Agne Tubeleviciute-Aydin, Andy P. Tsai, Andrew J. Saykin, P. Jesper Sjöström, Andrea C. LeBlanc

**Affiliations:** 1 Lady Davis Institute for Medical Research at Jewish General Hospital, Montreal, QC Canada; 2 Department of Anatomy and Cell Biology, McGill University, Montreal, QC Canada; 3 Department of Radiology and Imaging Sciences, Indiana Alzheimer’s Disease Research Center, Indiana University School of Medicine, Indianapolis, IN USA; 4 Centre for Research in Neuroscience, Department of Medicine, The BRaIN Program, Department of Neurology and Neurosurgery, The Research Institute, McGill University Health Centre, Montreal General Hospital, McGill University, 1650 Cedar Avenue Montreal, Montreal, QC H3G 1A4 Canada; 5 Department of Neurology and Neurosurgery, McGill University, Montreal, QC Canada; 6 Stark Neurosciences Research Institute, Indiana University School of Medicine, Indianapolis, IN USA

Correction to: *Scientific Reports* 10.1038/s41598-021-91367-0, published 16 June online 2021

The original version of this Article contained an error in Fig. 7g where the density maps in panel (g) were rendered incorrectly due to an error in the custom code. The different cell morphologies were not aligned properly, giving rise to a duplicated image and a widened impression in Fig. 7g. The original Fig. 7 and accompanying legend appear below.

**Figure 7:**
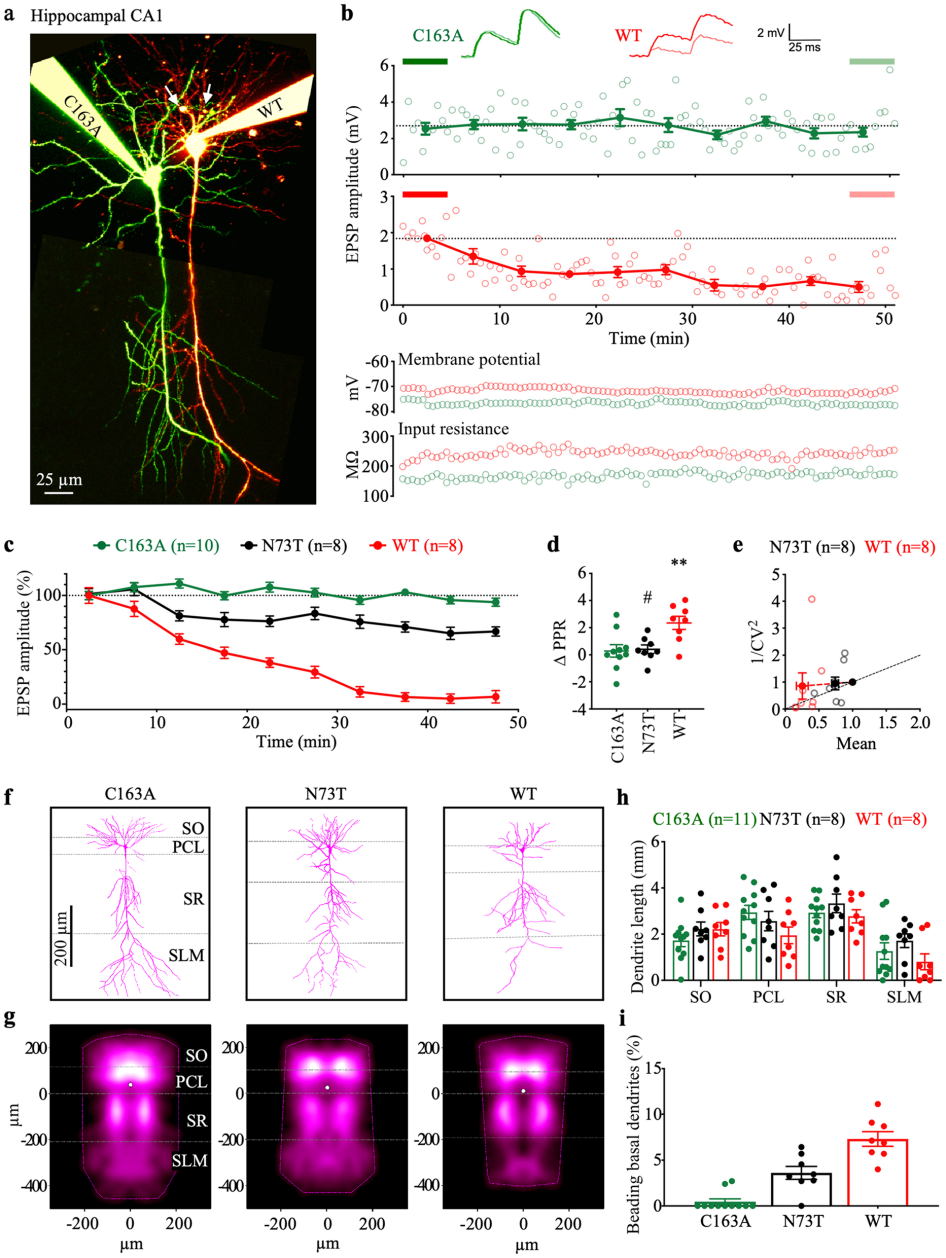
Casp6N73T is less damaging to neuronal function and neurodegeneration than Casp6WT. (**a**) Representative two-photon images of 10 pg Casp6C163A- (Alexa 488, green) and 10 pg Casp6WT- or Casp6N73T- (Alexa 594, red) patched hippocampal CA1 pyramidal neurons. White arrows indicate basal dendrite degeneration. Scale bar: 25 µm. Maximum-intensity projection of two-photon stacks was compiled using ImageJ, and imaging montage of entire neurons was performed using Affinity Designer 1.7. (**b**) Sample EPSP time course plots from Casp6C163A- (green) and Casp6WT- (red) patched neurons in (**a**), showing reduction of neurotransmission for Casp6WT (1.53 ± 0.32 mV, n = 8 vs. 0.14 ± 0.07 mV, n = 8, *p* < 0.01) but not for Casp6C163A (1.94 ± 0.32 mV, n = 10 vs. 1.91 ± 0.29 mV, n = 10, *p* = 0.76) when comparing the last 10 traces (light thick line) to the first 10 traces (dark thick line). Open circles: EPSP amplitude recorded every 30 s. Closed circles: EPSP amplitude binned and averaged across 10 traces. Inset: representative EPSP traces highlight the paired-pulse ratio (PPR). Scale bars: 2 mV, 25 ms. Resting membrane potential and input resistance remained stable throughout experiment. (**c**) EPSP time courses for Casp6C163A- (n = 10), Casp6N73T- (n = 8), or Casp6WT- (n = 8) patched neurons. (**d**) PPR from Casp6WT-, Casp6C163A- or Casp6N73T-patched neurons. One-way ANOVA (*p* = 0.0043), followed by Tukey’s post-hoc test (**p < 0.01 vs. C163A; ^#^*p* < 0.05 vs. WT). (**e**) CV analysis of Casp6N73T- and Casp6WT-patched neurons. Casp6C163A was unaltered. (**f**) Representative reconstructions of Casp6C163A-, Casp6N73T-, or Casp6WT-patched neurons. Image stacks were used for manual reconstruction of 3D morphologies using the Neuromantic freeware (http://www.reading.ac.uk/neuromantic/body_index.php). (**g**) Dendritic density maps of Casp6C163A- (n = 11), Casp6N73T- (n = 8), or Casp6WT- (n = 8) patched neurons generated using custom software running in Igor Pro 8 v8.04 (https://www.wavemetrics.com, https://github.com/pj-sjostrom/qMorph). Dotted lines show the convex hull of the maximum extent. (**h**) Cumulative dendritic length of reconstructed neurons in layers SO, PCL, SR, and SLM. (**i**) Casp6C163A-, Casp6N73T- and Casp6WT-patched CA1 pyramidal neurons beading basal dendrites. One-way ANOVA (*p* = 0.0001), followed by Tukey’s test (****p < 0.0001 vs Casp6C163A, ^##^*p* < 0.01 vs Casp6WT).

The original Article has been corrected.

