# Supplementary material for: Rare CASP6N73T variant associated with hippocampal volume exhibits decreased proteolytic activity, synaptic transmission defect, and neurodegeneration
Source: Sci Rep. 2021 Jun 16;11:12695. doi: 10.1038/s41598-021-91367-0 (PMC8209045; doi:10.1038/s41598-021-91367-0)
Supplement: Supplementary file 1 — Supplementary Information. [file 41598_2021_91367_MOESM1_ESM.pdf]

SUPPLEMENTAL INFORMATION

Rare *CASP6*N73T variant associated with hippocampal volume exhibits altered proteolytic activity, synaptic transmission defect and neurodegeneration

Libin Zhou[1,2], Kwangsik Nho[3], Maria G. Haddad[4], Nicole Cherepacha[4], Agne Tubeleviciute-Aydin[1,5], Andy P. Tsai[6], Andrew J. Saykin[3], P. Jesper Sjöström[4], and Andrea C. LeBlanc[1,2,5*]; Alzheimer's Disease Neuroimaging Initiative (ADNI) [¥]

[1]Lady Davis Institute for Medical Research at Jewish General Hospital, Montréal, Québec, Canada
[2]Department of Anatomy and Cell Biology, McGill University, Montréal, Québec, Canada
[3]Department of Radiology and Imaging Sciences and Indiana Alzheimer Disease Center, Indiana University School of Medicine, Indianapolis, IN, USA
[4]Centre for Research in Neuroscience, the BRaIN Program, Department of Medicine, and Department of Neurology and Neurosurgery, McGill University, The Research Institute of the McGill University Health Centre, Montreal General Hospital, 1650 Cedar Avenue Montreal, QC H3G 1A4, Canada
[5]Department of Neurology and Neurosurgery, McGill University, Montréal, Québec, Canada
[6]Stark Neurosciences Research Institute, Indiana University School of Medicine, Indianapolis, IN, USA

*Corresponding author: Andrea LeBlanc, PhD, Bloomfield Center for Research in Aging, Lady Davis Institute for Medical Research, Sir Mortimer B Davis Jewish General Hospital, 3755 ch. Côte Ste-Catherine, Montréal, QC, Canada H3T 1E2. Tel.: +1 (514) 340 8222 ext 24976. e-mail address: andrea.leblanc@mcgill.ca

[¥]Data used in preparation of this article were obtained from the Alzheimer's Disease Neuroimaging Initiative (ADNI) database (http://adni.loni.usc.edu). As such, the investigators within the ADNI contributed to the design and implementation of ADNI and/or provided data but did not participate in analysis or writing of this report. A complete listing of ADNI investigators can be found at: http://adni.loni.usc.edu/wp-content/uploads/how_to_apply/ADNI_Acknowledgement_List.pdf.

Supplemental figure S1 Zhou et al., A rare Casp6 variant associated with hippocampal volume

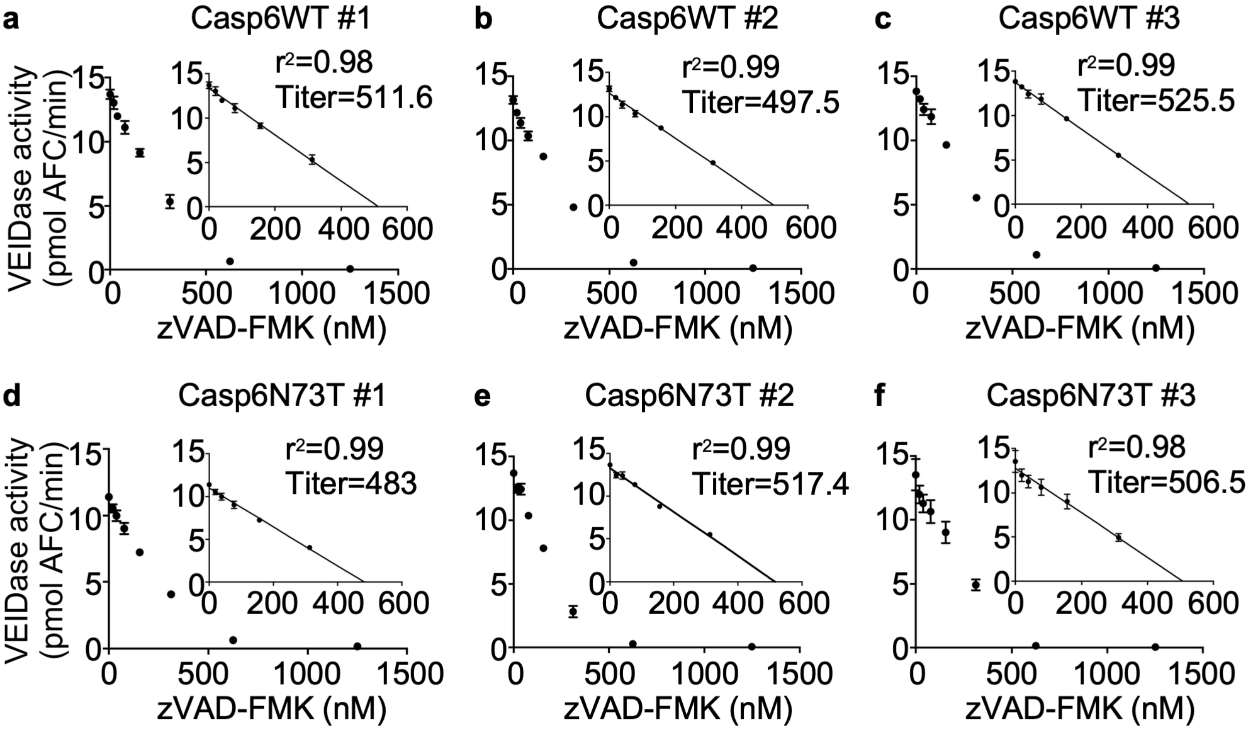

Supplemental figure S1. **Active site titration of purified recombinant Casp6WT and Casp6N73T. (a-f)** Active site titration of recombinant Casp6WT #1 (520 nM) (**a**), Casp6WT #2 (502 nM) (**b**), Casp6WT #3 (525 nM) (**c**), Casp6N73T #1 (464 nM) (**d**), Casp6N73T #2 (544 nM) (**e**), and Casp6N73T #3 (503 nM) (**f**) using irreversible inhibitor zVAD-FMK. The inlets of the graphs show linear regression of data points corresponding to 0-1250 nM of zVAD-FMK, the x-axis intercept (titer) is equal to the active caspase concentration in the titration assay, showing the % active site is 98.4% for Casp6WT #1, 99.1% for Casp6WT #2, 100% for Casp6WT #3, 104% for Casp6N73T #1, 95.3% for Casp6N73T #2, and 100% for Casp6N73T #3. Obtained titer values indicate that purified recombinant caspases are more than 95% active. The data points represent mean and s.e.m. from three independent experiments.

Supplemental figure S2 Zhou et al., A rare Casp6 variant associated with hippocampal volume

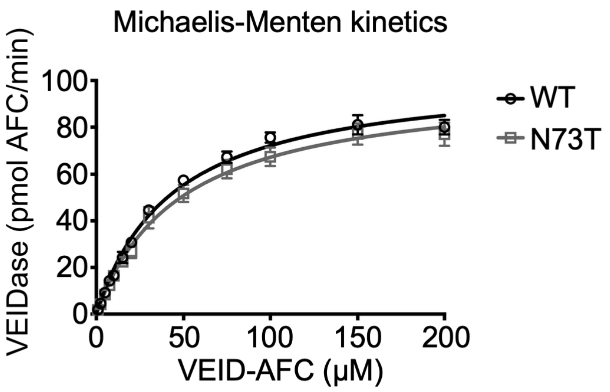

Supplemental figure S2. **Michaelis-Menten kinetics of recombinant Casp6N73T compared to Casp6WT.** The reaction velocity (VEIDase) of 20 nM active-site titrated Casp6WT or Casp6N73T on 1-200 µM Ac-VEID-AFC. Data were fitted into Michaelis-Menten equation using nonlinear regression and represent the mean ± sem from three independent experiments.

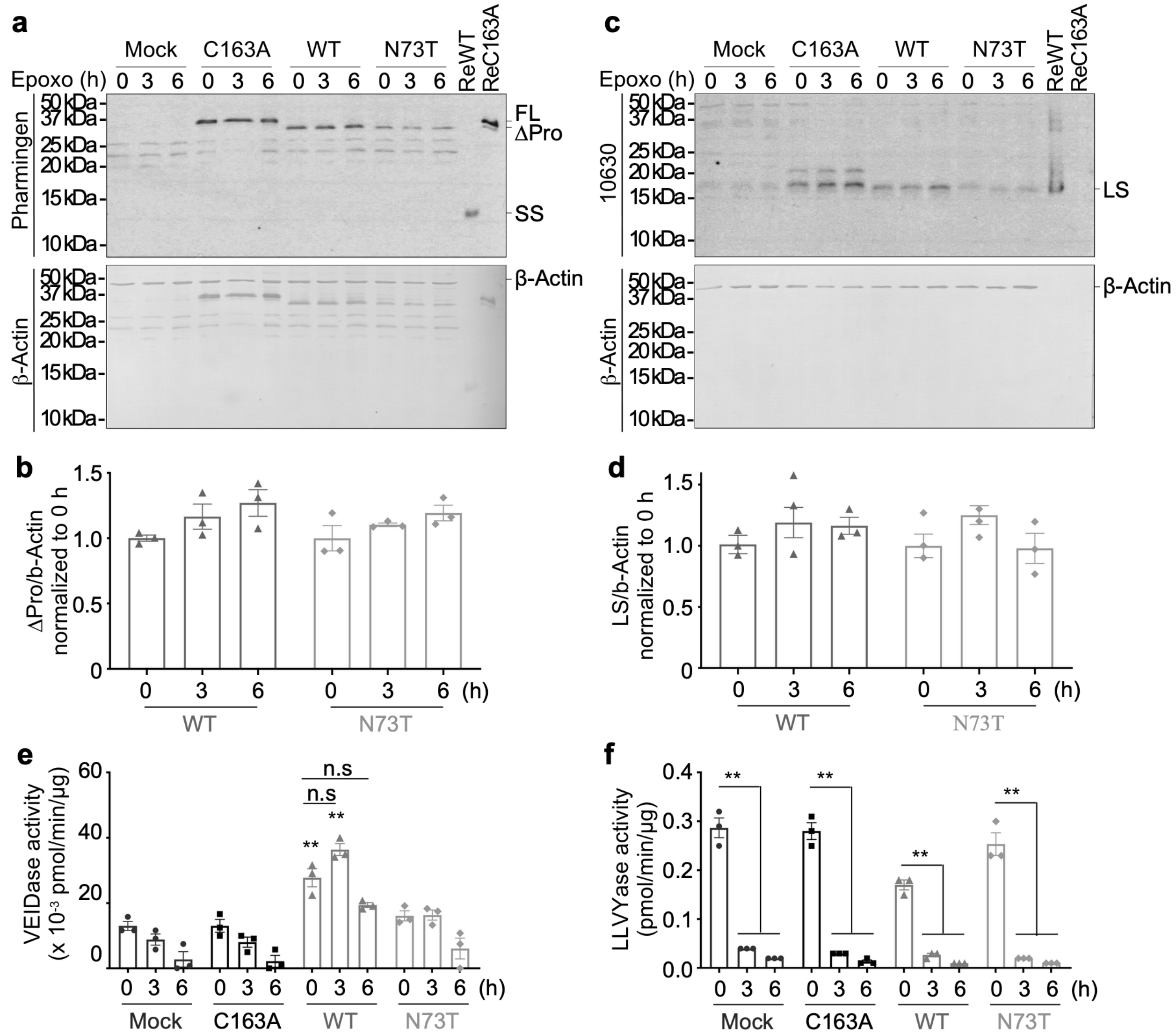

Supplemental figure S3. **The proteasome inhibitor epoxomicin did not change the level or activity of Casp6N73T in HEK293T cells.** (**a&c**) Western blot and (**b&d**) quantification of Casp6 in transfected cells against Pharmingen (**a&b**) or 10630 neoepitope antibodies (**c&d**). Epoxo, epoxomicin; ReWT, recombinant Casp6WT; ReC163A, recombinant Casp6C163A; FL, full length; ΔPro: Casp6 without pro-domain; SS, small subunit; LS, large subunit. No statistical differences were found by one-way ANOVA. (**e**) VEIDase activity in protein extract from transfected cells. Statistical evaluations were done with one-way ANOVA ($p < 0.01$) followed by post-hoc Tukey's test. **p < 0.01 vs Mock at 0 h. (**f**) Inhibition of proteasome activity was confirmed by measuring LLVYase activity. Statistical evaluations were done with one-way ANOVA ($p < 0.01$) followed by post-hoc Tukey's test. **p < 0.01.

Supplemental figure S4 Zhou et al., A rare Casp6 variant associated with hippocampal volume

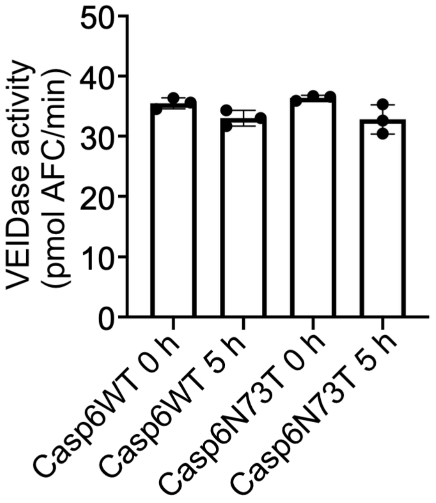

Supplemental figure S4. **The stability of active recombinant Casp6N73T in internal solution.** VEIDase assay indicates that activity of recombinant Casp6WT and Casp6N73T did not change significantly in internal solution within 5 hours.

Western blot originals for Figure 4a

Figure 4a

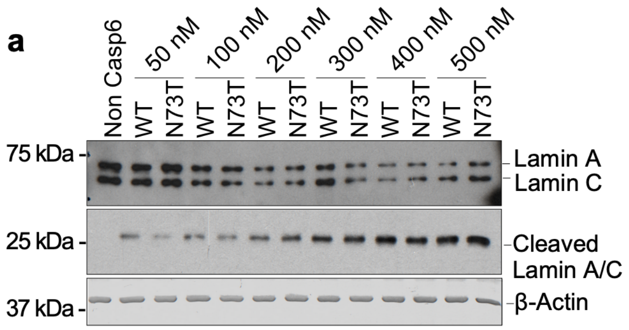

Western blot originals for Figure 4a

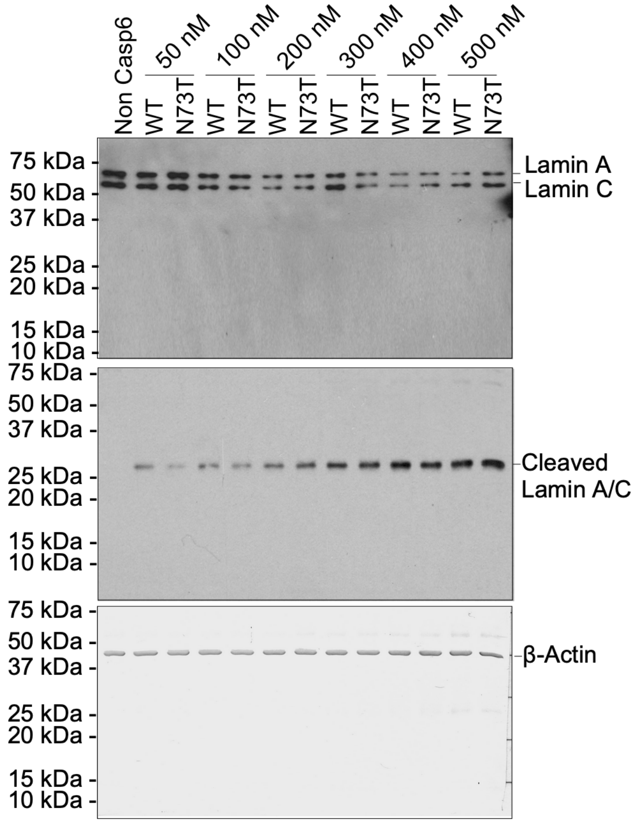

Western blot originals for Figure 4c

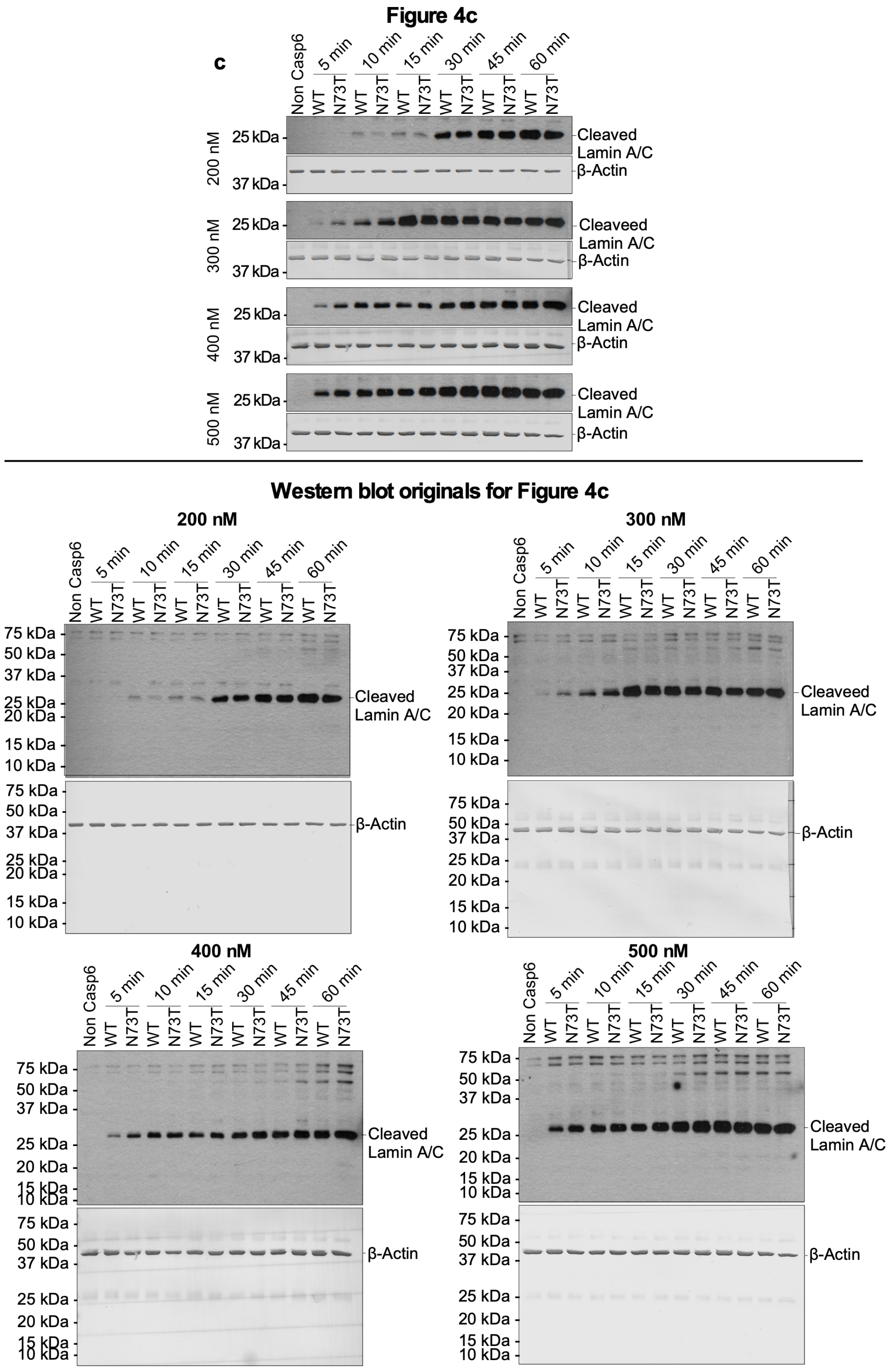

Western blot originals for Figure 5a

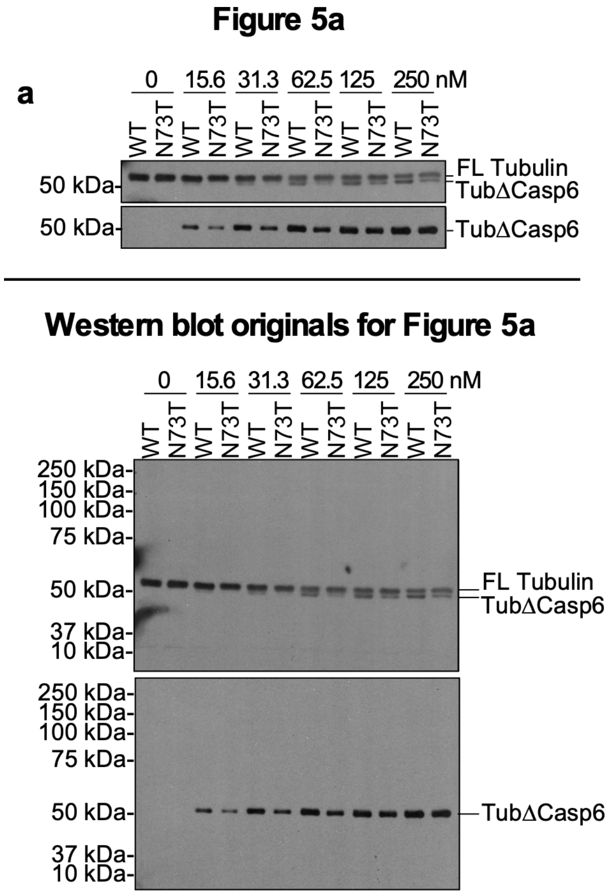

Western blot originals for Figure 5c

Figure 5c

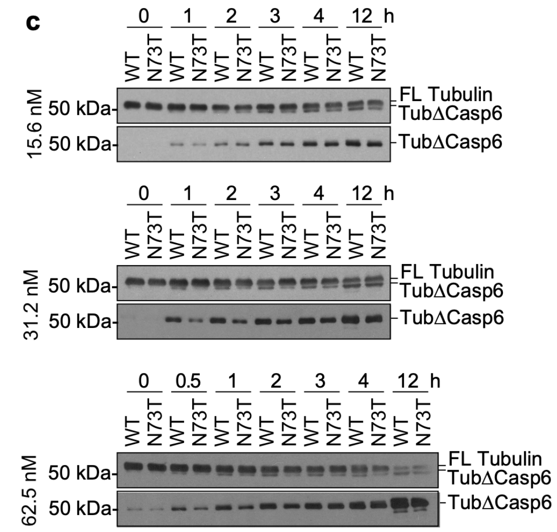

Western blot originals for Figure 5c

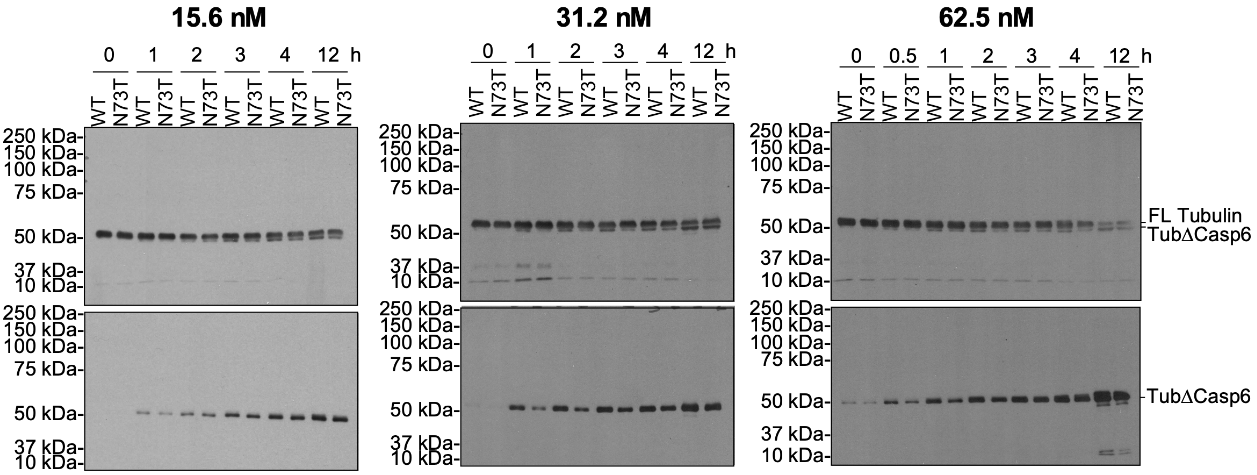

Original agarose gels for Figure 6a

Figure 6a

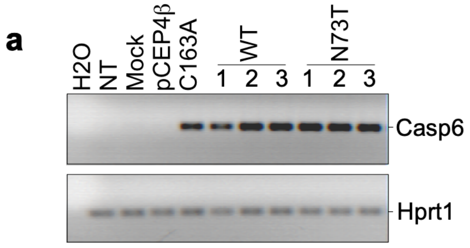

Original gels of Figure 6a

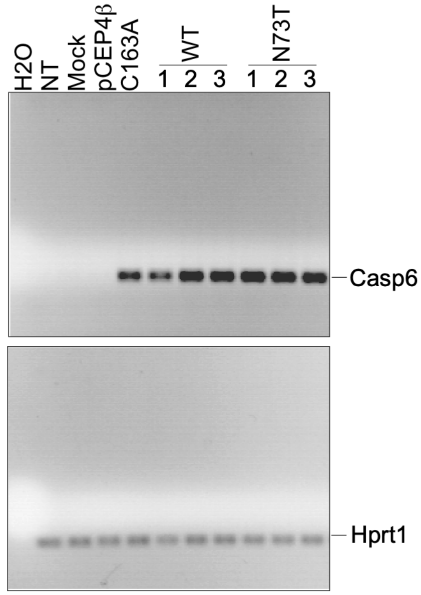

Western blot originals for Figure 6c&e

Figure 6c&e

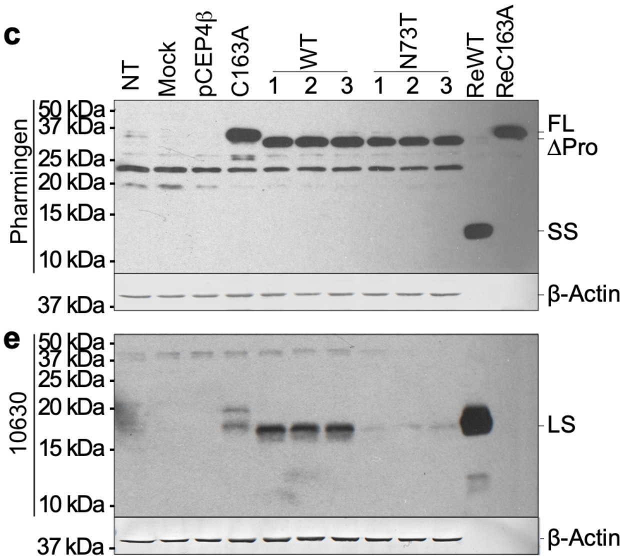

Western blot originals for Figure 6c&e

Pharmingen

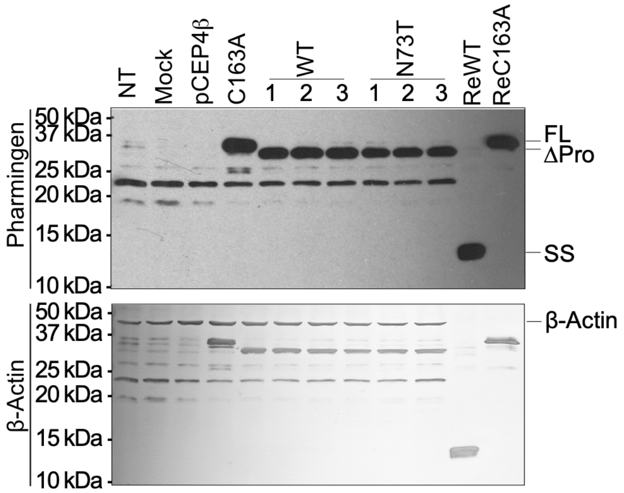

10630

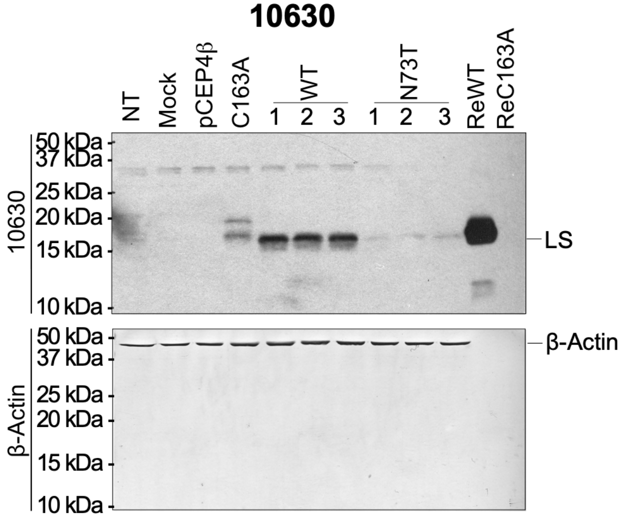

136 *Western blot originals for Figure 6h*

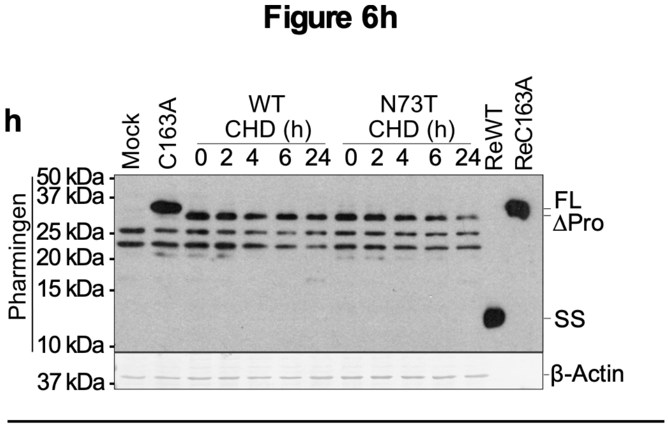

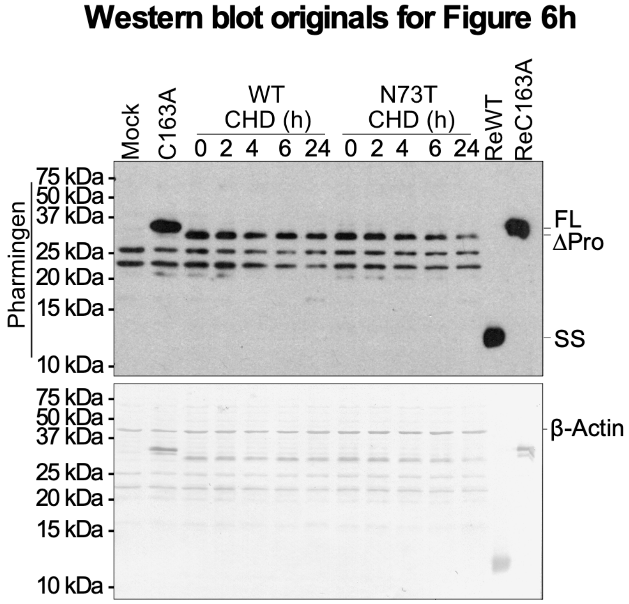

137

138
